# Association between results of component-resolved diagnostics and basophil activation in Hymenoptera venom allergy: A registry-based cross-sectional study in adults

**DOI:** 10.1371/journal.pone.0350189

**Published:** 2026-06-17

**Authors:** Krzysztof Łukasz Piwowarek, Krzysztof Kłos, Elżbieta Rutkowska, Andrzej Chciałowski, Agata Raniszewska-Borys, Iwona Kwiecień, Jerzy Kruszewski

**Affiliations:** 1 Department of Internal Medicine, Infectious Diseases and Allergology, Military Institute of Medicine – National Research Institute, Warsaw, Poland‌‌; 2 Department of Battlefield Medicine, Military Institute of Medicine – National Research Institute, Warsaw, Poland; 3 Laboratory of Hematology and Flow Cytometry, Military Institute of Medicine – National Research Institute, Warsaw, Poland; Charles University: Univerzita Karlova, CZECHIA

## Abstract

**Background:**

Allergy to *Hymenoptera* venom is one of the most frequent causes of anaphylaxis in adults. Conventional diagnostic approaches, including skin testing and measurement of allergen-specific IgE (sIgE) to venom extracts, do not always allow for precise identification of the culprit venom. This remains particularly challenging in patients with double-positive or inconclusive conventional test results, which may complicate the decision-making process regarding qualification for appropriate treatment. Component-resolved diagnostics (CRD) and the basophil activation test (BAT) may provide complementary information in such cases and aid in qualification for subcutaneous immunotherapy (SCIT).

**Methods:**

In this retrospective registry-based cross-sectional study, 154 adults who had been evaluated for *Hymenoptera* venom allergy at the Military Institute of Medicine (Warsaw, Poland) between December 2023 and May 2025 were included. Patients were divided into three groups: not qualified for SCIT (n = 27), qualified for bee venom immunotherapy (n = 32), and qualified for wasp venom immunotherapy (n = 95). Serum sIgE to venom extracts and certain components (rApi m 1, m 2, m 3, m 5, m 10; rVes v 1, v 5) were measured. BAT was performed by flow cytometry assessing CD63 expression.

**Results:**

Moderate correlations were found between BAT results and sIgE to rApi m 1 (rho = 0.495; p < 0.001) and rVes v 5 (rho = 0.456; p < 0.001). No correlation was observed for rVes v 1, and negative correlations were noted between rApi m 1 and rVes v 5 in heterologous BAT responses. No statistically significant association was observed between the severity of previous sting reactions and BAT or sIgE parameters. However, due to limited subgroup sizes, these analyses were underpowered and the results should be interpreted as inconclusive.

**Conclusions:**

Among the examined variables, the major components Api m 1 and Ves v 5 were the most strongly correlated with specific basophil activation in vitro. The combined use of CRD and BAT may support clinical assessment and facilitate decisions regarding immunotherapy qualification. Further prospective studies are warranted to assess the prognostic and clinical relevance of our findings.

## Introduction

Allergy to *Hymenoptera* venom, particularly from bees and wasps, is one of the most common causes of anaphylaxis in adults in European countries [1–3]. The estimated risk of an anaphylactic reaction following a sting ranges from 0.3% to 3% in the general population, while the prevalence of IgE sensitization may reach up to 25% [4]. This emphasizes the clinical and public health importance of this condition, especially considering the potential for life-threatening anaphylactic shock. Moreover, climate change may influence the geographical distribution of *Hymenoptera* species and thereby alter the risk of stings, including those from less common species such as paper wasps (*Polistes* spp.). Fortunately, an effective causal treatment is available — venom immunotherapy (VIT). However, this therapy typically lasts five years and is associated with substantial pharmacoeconomic costs as well as certain inconveniences for patients. Therefore, careful and well-considered qualification for this treatment remains of paramount importance.

In light of these challenges, diagnostic approaches based on patient history, conventional skin testing, and measurements of allergen specific IgE (sIgE) to whole allergen extracts do not always allow for unambiguous identification of the allergy source. Consequently, component-resolved diagnostics (CRD) and the basophil activation test (BAT) are gaining increasing interest [5,6].

Measurement of IgE specific to native or recombinant protein allergens, such as rApi m 1 (*Apis mellifera*) or rVes v 5 (*Vespula vulgaris*), allows for precise identification of the sensitizing source, particularly in patients with positive results to multiple extracts or with an inconclusive clinical history [7]. Concurrently, the basophil activation test, which assesses the functional in vitro response of basophils to an allergen by measuring the expression of activation surface markers (e.g., CD63), provides valuable complementary information [8–9]. This is particularly important in the context of qualification for subcutaneous immunotherapy (SCIT), which is recommended for patients with a confirmed diagnosis of *Hymenoptera* venom allergy who have experienced systemic reactions following a sting. The combined use of BAT and CRD may potentially help confirm the diagnosis and facilitate the decision-making process, particularly in cases with double-negative or double-positive results, which still remain challenging. The integrative diagnostic approach is especially valuable for patients requiring concomitant SCIT with both honeybee and wasp venom.

The primary objective of this retrospective registry-based cross-sectional study was to determine the relationship between the profile of sIgE to venom components and the results of BAT in three groups of patients: those not qualified for SCIT due to the absence of confirmed venom allergy (group A), those qualified for bee venom SCIT (group B), and those qualified for wasp venom SCIT (group C). The secondary objective was to analyze the association between the investigated parameters and the severity of anaphylactic reactions in the medical history of participants.

## Materials and methods

### Setting and study participants

This retrospective, registry-based cross-sectional analysis included 154 adult patients evaluated for *Hymenoptera* venom allergy at the Department of Internal Medicine, Infectious Diseases and Allergology, Military Institute of Medicine in Warsaw, from the beginning of December 2023 to the end of May 2025. All participant data were anonymized prior to analysis. Data extraction, aggregation and analysis were conducted between November 1–20, 2025, after obtaining approval from the Bioethics Committee.

It should be noted that patients with ongoing acute infections, pregnant women, and individuals unable to provide informed consent to routine clinical diagnostic procedures or venom immunotherapy were excluded from the study. Patients were evaluated according to a stepwise diagnostic approach based on routine clinical practice:

1. A routine medical history was obtained from all participants, with particular emphasis on previous reactions to insect stings, comorbidities, and current medications. The severity of prior anaphylactic reactions was assessed using the four-grade Müller scale [10].
2. Initial assessment of sensitization to *Hymenoptera* venom was performed using serum sIgE measurements.
3. Qualification for venom immunotherapy and assignment to the bee or wasp group were based on clinical history consistent with systemic reaction and evidence of sensitization to a given venom. Sensitization was considered documented if the patient had a positive result for venom extract-specific IgE, or for component-resolved diagnostics (Api m 1, Api m 2, Api m 3, Api m 10, Ves v 1, or Ves v 5).
4. The basophil activation test was performed as a complementary investigation and was not used as a primary criterion for group assignment.

The study participants were divided into three groups according to the final diagnosis and qualification for treatment. Group A consisted of patients who were not diagnosed with venom allergy, defined as the absence of venom-specific IgE antibodies and a negative basophil activation test. The threshold for sIgE was 0.35 IU/mL.

Group B consisted of patients who were qualified for bee venom immunotherapy, and group C consisted of patients who were qualified for wasp venom immunotherapy. Additionally, in groups B and C, two subcategories of patients were distinguished based on the severity of the most severe anaphylactic reaction in the medical history:

- Patients with mild reactions: large local reactions and Müller grade I-II anaphylaxis.
- Patients with severe reactions: Müller grade III–IV anaphylaxis.

This approach was intended to enable the assessment of associations between BAT and CRD results and the severity of allergic symptoms.

### Laboratory procedures

As part of the standard diagnostic protocol, peripheral venous blood samples were collected from all study participants. The following parameters were measured in the collected samples: complete blood count with automated smear, serum tryptase concentration, allergen specific IgE to honeybee and wasp venom extracts, and sIgE to individual molecules: rApi m 1, m 2, m 3, m 5, m 10 (*Apis mellifera*) and rVes v 1, v 5 (*Vespula vulgaris*) listed in Table 1. Complete blood counts were performed using an XN-1000 analyzer (Sysmex, Japan). Measurements of serum tryptase and sIgE concentrations were performed using the ImmunoCAP method (Phadia/Thermo Fisher Scientific, Sweden). IgE testing was performed according to the current availability of assays.

**Table 1:** The list of *Hymenoptera* allergens included in the study. Nomenclature according to WHO/IUIS Allergen Nomenclature Sub-Committee [11].

Allergen according to nomenclature	Biochemical name	Molecular mass [kDa]	Allergen source
Api m 1	Phospholipase A2	16	*Apis mellifera*
Api m 2	Hyaluronidase	44	
Api m 3	Acid phosphatase	43	
Api m 5	Dipeptidylpeptidase IV	100	
Api m 10	Icarapin	50-55	
Ves v 1	Phospholipase A1B	34	*Vespula vulgaris*
Ves v 5	Antigen 5	23	
Pol d 5	Antigen 5	23	*Polistes dominulus*

In addition, the basophil activation test was performed using the commercial BasoFlowEx Kit (EXBIO Praha a.s., Czech Republic). Blood samples for BAT were collected into sodium heparin tubes (100 µL per sample). The samples were incubated with the following reagents: control buffer (negative control), stimulating solution containing anti-IgE antibodies and formyl-methionyl-leucyl-phenylalanine (fMLP) as a positive control, honeybee venom at the concentration of 10 µg/mL, and wasp venom at the similar concentration. Venom solutions were prepared from commercially available extracts, Venomenhal biene and Venomenhal wespe (HAL Allergy, the Netherlands). The incubation consisted of two steps: 25 minutes in an air incubator at 37°C, followed by 20 minutes at 2–8 °C.

Staining was performed as a subsequent step after stimulation. Twenty µL of staining reagent was added to each tube. Samples were then incubated for 20 minutes at 2–8 °C. No stopping solution was used. Immediately after the staining step, lysing solution was added. After 5 minutes – once red blood cells were lysed – the samples were centrifuged, the supernatant was removed, and the pellet was resuspended in 200 µL of PBS (Phosphate Buffered Saline). The samples were then analyzed immediately using a flow cytometer. At least 200 basophils per sample were analyzed, which represents the minimum acquisition threshold ensuring reliability of the BAT results.

Basophils were identified as CD203c ⁺ SSC low cells and their activation was assessed based on the induced expression of CD63. A positive BAT result was defined as ≥10% CD63 ⁺ basophils, provided that at least 20% activation was observed in the positive control.

Approximately 10–15 mL of peripheral venous blood was collected from each participant to perform all laboratory analyses described above.

### Ethical approval

All procedures were conducted in accordance with the ethical standards of the institutional and national research committees and with the principles of the Declaration of Helsinki. The study protocol was reviewed and approved by the Bioethics Committee of the Military Institute of Medicine – National Research Institute (Warsaw, Poland; approval No. 43/25, issued on September 17, 2025). The approval covered retrospective analysis of already collected data. The requirement for written informed consent was waived by the Bioethics Committee, as the study was retrospective and conducted on anonymized data.

### Statistical analysis

Statistical analysis was performed using the open-source software JAMOVI, version 2.3. The normality of continuous variables was assessed with the Shapiro–Wilk test, which in the vast majority of cases indicated non-normal distributions. Accordingly, comparisons among the three study groups were conducted using the Kruskal–Wallis ANOVA. When appropriate, post hoc analyses (Dwass–Steel–Critchlow–Fligner, DSCF) were applied based on the global test results. Given the exploratory nature of the study, no additional adjustment for multiple testing was applied beyond the DSCF procedure and additional correction (e.g. Bonferroni’s) was not needed.

For comparisons between the two anaphylaxis severity categories, the Mann–Whitney U test was used. Categorical variables were compared using the χ² test.

Correlations between individual variables were assessed using Spearman’s rank correlation. Due to the exploratory design of the study, p-values were interpreted descriptively and without formal adjustment for multiple comparisons. The strength of correlation was interpreted according to following criteria: rho < 0.2 – very weak or no correlation, 0.2–0.39 – weak, 0.4–0.59 – moderate, 0.6–0.79 – strong, and ≥ 0.8 – very strong. A p value < 0.05 was considered statistically significant [12–13].

Regarding the number of statistical comparisons performed, all analyses should be considered exploratory in nature. The reported p-values are presented as descriptive measures of association and should be interpreted as hypothesis-generating rather than confirmatory. Therefore, results, particularly those with p-values close to the conventional significance threshold, should be interpreted with caution.

## Results

### Population characteristics

A total of 154 consecutive patients presenting to the Department were included in the study: 27 in the group not qualified for SCIT due to the absence of confirmed venom allergy (group A), 32 in the group qualified for bee venom SCIT (group B), and 95 in the group qualified for wasp venom SCIT (group C). One case of a double-sensitized patient was classified into Group B, as wasp venom allergy was considered the primary cause of anaphylaxis in this case and constituted the target of immunotherapy.

The groups were relatively homogeneous in terms of demographic parameters; in each group, the mean age was approximately 50 years (Table 2), and sex distribution was comparable (χ² = 2.15; df = 2; p = 0.342). Similarly, as expected, there were no significant differences in basic laboratory findings, such as peripheral blood leukocyte count and serum tryptase concentration.

**Table 2:** Measurement results in the study groups.

Variable	Group A			Group B			Group C			ANOVA K-W^d^ – p	DSCF^e^ post hoc p		
	Mean (95% CI^a^)	SD ^b^	Med^c^	Mean (95% CI^a^)	SD ^b^	Med^c^	Mean (95%CI^a^)	SD ^b^	Med^c^		0 vs 1	0 vs 2	1 vs 2
Females [count]	n = 19 [70.4 %]			n = 18 [56.3 %]			n = 52 [54.7 %]			N/A	N/A		
Males [count]	n = 8 [29.6 %]			n = 14 [43.7 %]			n = 43 [45.3 %]						
Age [years]	50.3 (±5.88)	15.6	50.0	47.9 (±5.51)	15.9	45.5	50.5 (±2.45)	12.2	49.0	0.632	N/A		
Neutrophils [count]	3560 (±475)	1260	3440	3790 (±429)	1240	3630	3640 (±255)	1270	3540	0.844			
Basophils [count]	36 (±6.04)	16	30	66 (±47.81)	138	30	45 (±6.44)	32	40	0.273			
Eosinophils [count]	146 (±39.60)	105	140	163 (±39.85)	115	130	135 (±16.89)	84	120	0.654			
Tryptase [µg/L]	5.95 (±1.61)	4.28	4.8	6.57 (±0.82)	2.37	6.04	8.34 (±1.30)	6.48	5.71	0.170			
BAT control pos.^f^ [%]	50.6 (±10.90)	28.9	54.3	53.0 (±8.04)	23.2	52.7	62.2 (±4.48)	22.3	64.7	**0.046**	0.984	0.112	0.137
BAT control neg.^g^ [%]	2.72 (±1.05)	2.78	1.8	1.49 (±0.54)	1.55	1.2	2.79 (±1.18)	5.87	1.11	0.167	N/A		
BAT bee [%]	8.85 (±4.98)	13.2	3.5	42.9 (±11.88)	34.3	50.2	6.2 (±2.73)	13.6	1.5	**<0.001**	**0.002**	**0.025**	**<0.001**
BAT wasp [%]	8.4 (±3.96)	10.5	4.6	7.58 (±4.92)	14.2	2.45	45.3 (±5.67)	28.2	51.6	**<0.001**	0.176	**<0.001**	**<0.001**
sIgE bee [IU/mL]	0.762 (±0.68)	1.79	0.02	16.4 (±8.21)	23.7	9.03	0.718 (±0.51)	2.53	0.02	**<0.001**	**<0.001**	0.894	**<0.001**
sIgE wasp [IU/mL]	0.365 (±0.24)	0.646	0.15	1.72 (±1.37)	3.95	0.175	10.1 (±3.64)	18.1	3.77	**<0.001**	0.712	**<0.001**	**<0.001**
rApi m 1 [IU/mL]	0.088 (±0.07)	0.188	0	7.22 (±4.96)	14.3	1.27	0.214 (±0.36)	1.77	0	**<0.001**	**<0.001**	0.342	**<0.001**
rApi m 2 [IU/mL]	0.082 (±0.11)	0.300	0	2.31 (±1.44)	4.15	0.1	0.163 (±0.14)	0.678	0	**<0.001**	**0.002**	0.834	**<0.001**
rApi m 3 [IU/mL]	0.029 (±0.03)	0.074	0	0.816 (±0.87)	2.51	0.08	0.052 (±0.044)	0.22	0	**<0.001**	**<0.001**	0.813	**<0.001**
rApi m 5 [IU/mL]	0.163 (±0.24)	0.644	0	0.841 (±0.96)	2.76	0.03	0.850 (±0.77)	3.82	0	**0.01**	**0.014**	0.376	**0.049**
rApi m 10 [IU/mL]	0.144 (±0.15)	0.402	0	3.31 (±1.93)	5.58	0.495	0.088 (±0.064)	0.319	0	**<0.001**	**<0.001**	0.996	**<0.001**
rVes v 1 [IU/mL]	0.351 (±0.42)	1.11	0.02	0.566 (±0.59)	1.71	0.055	2.25 (±1.54)	7.64	0.085	**0.032**	0.634	**0.042**	0.273
rVes v 5 [IU/mL]	0.325 (±0.29)	0.773	0.13	0.399 (±0.53)	1.53	0.04	9.12 (±3.54)	17.6	2.34	**<0.001**	0.853	**<0.001**	**<0.001**

^a^Condifdence interval ^b^ Standard deviation ^c^ Median; ^d^ Kruskal-Wallis; ^e^ Dwass–Steel–Critchlow–Fligner; ^f^ Positive; ^g^ Negative

Missing data were limited and are presented in the Supporting Information (S3 Table). Complete-case analyses yielded results consistent with those obtained in the full analytic sample. All other parameters were complete for the analyzed cohort. A normal distribution was observed only for age in Groups A and B and for peripheral blood neutrophil count in Group A. All other variables deviated from normality. Hence, nonparametric tests were applied throughout the analysis.

### Allergen specific IgE immunoglobulin levels

Statistically significant differences were observed in serum concentrations of IgE specific to honeybee venom extract and certain honeybee venom components between Group A and Group B, as well as between Group B and Group C, consistent with expectations. A similar pattern was found for IgE specific to wasp venom extract and its components, with significant differences between Groups A and C and between Groups B and C. The only exception was IgE directed against rVes v 1, for which no statistically significant difference was observed between patients qualified for bee venom SCIT and those qualified for wasp venom SCIT in our study population.

### Basophil activation test

**Fig 1:**
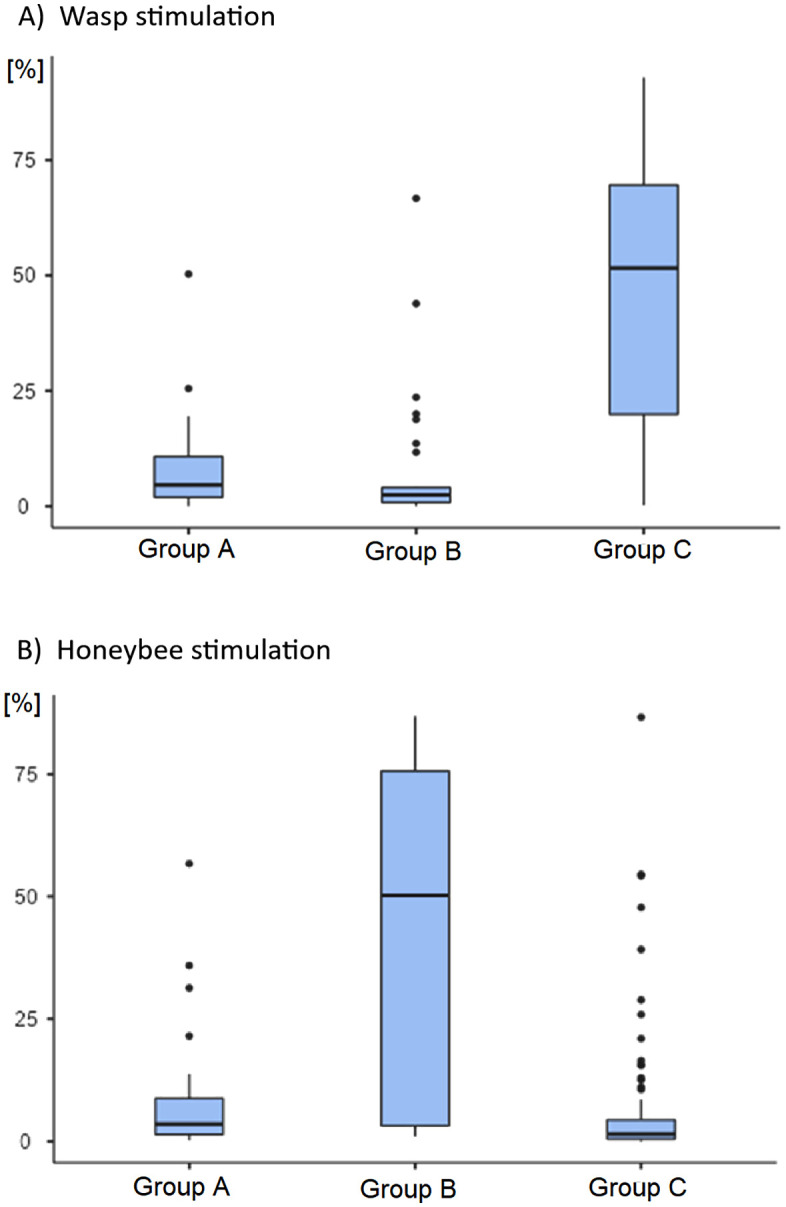
Basophil activation (CD63 ⁺ , %) induced by wasp venom and honeybee venom across the study groups A–C.

As expected, basophil reactivity was significantly higher for the venom of culprit insect (Fig 1). In group B, the mean BAT response to honeybee venom was 42.9% (p < 0.001), while in group C it was 45.3% for wasp venom (p < 0.001). In contrast, spontaneous basophil reactivity assessed by the negative control did not differ significantly among the study groups, nor did nonspecific reactivity assessed by the positive control. Although the global test indicated a borderline difference (p = 0.046), post hoc analyses did not reveal statistically significant pairwise differences between the groups.

**Fig 2:**
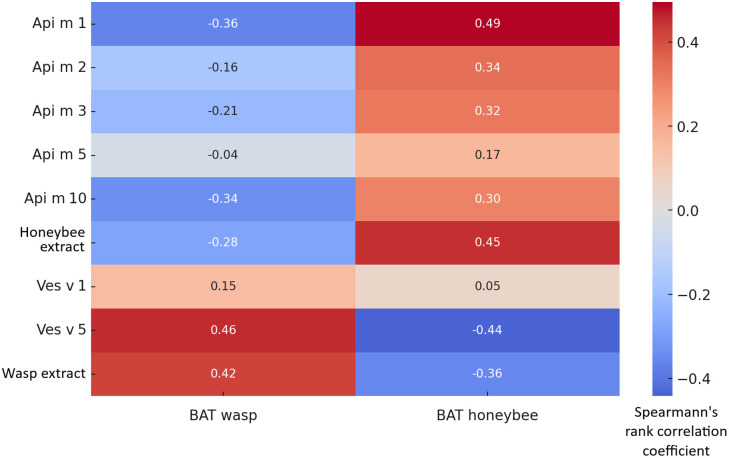
Four different patterns of the BAT results shown as two-dimensional histograms. Following lines from top to bottom: A) a patient reactive to honeybee venom; B) a patient reactive to wasp venom; C) a patient non-reactive to honeybee venom nor to wasp venom; D) a rare case of double-positive reactivity.

Most patients exhibited monosensitization profiles consistent with either honeybee or wasp venom allergy, as reflected by concordant component-resolved diagnostics and basophil activation test results. A distinct subgroup showed low or absent basophil reactivity despite detectable venom-specific IgE. Notably, in one patient a clear pattern of dual sensitization was identified, with simultaneous reactivity to both honeybee and wasp venom components, accompanied by corresponding basophil activation responses (Fig 2).

**Fig 3:**
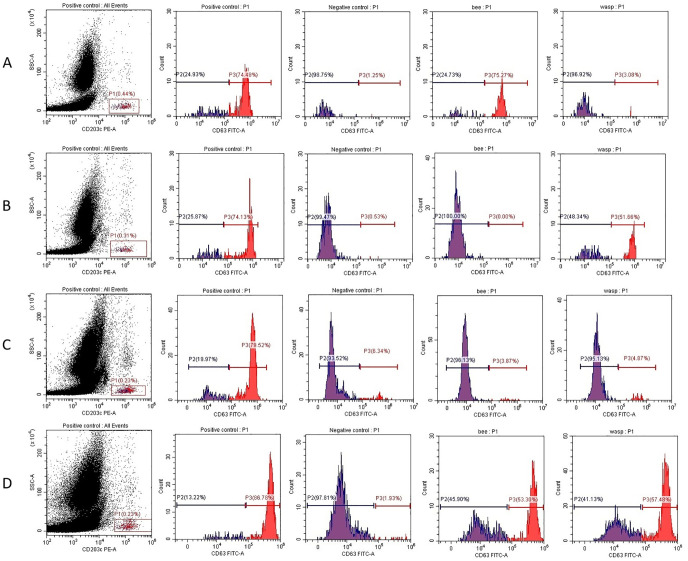
Heatmap of Spearman’s correlations (rho) between sIgE (extracts and components) and BAT responses (bee/wasp)‌‌.

Interestingly, BAT results for wasp venom correlated primarily with serum concentrations of IgE specific to rVes v 5 (rho = 0.456; p < 0.001), while no significant correlation was observed with rVes v 1. A similar pattern was noted for honeybee venom BAT results, which correlated mainly with IgE specific to rApi m 1 (rho = 0.495; p < 0.001) and rApi m 2 (rho = 0.339; p < 0.001), whereas correlations with other components were considerably weaker or, as in the case of rApi m 3, absent. Negative correlations were also observed, with the two strongest involving rApi m 1 and rVes v 5 in relation to BAT results obtained for the heterologous venom solution (Fig 3, Table 3).

**Table 3:** Correlations between basophil activation test results and allergen specific IgE immunoglobulin levels.

Variable	BAT results for wasp		BAT results for honeybee	
	rho	p	rho	p
rApi m 1	−0.365	**< 0.001**	**0.495**	**< 0.001**
rApi m 2	−0.162	**0.049**	0.339	**< 0.001**
rApi m 3	−0.212	**0.010**	0.322	**< 0.001**
rApi m 5	−0.038	0.650	0.165	**0.047**
rApi m 10	−0.337	**< 0.001**	0.299	**<0.001**
Honeybee extract	−0.280	**< 0.001**	**0.448**	**< 0.001**
rVes v 1	0.154	0.060	0.054	0.514
rVes v 5	**0.456**	**< 0.001**	**−0.442**	**< 0.001**
Wasp extract	**0.424**	**< 0.001**	−0.357	**< 0.001**

### IgE immunoglobulin specific to hornet venom extract and Pol d 5

IgE antibodies against hornet venom extract and the Pol d 5 (*Polistes dominulus*) component were measured in only 20 and 17 individuals from group A, 20 and 15 from group B, and 70 and 63 from group C, respectively. Therefore, these results were not included in the summary tables.

Nevertheless, for the hornet venom extract, the mean sIgE concentrations were 0.273 IU/mL in group A, 0.877 IU/mL in group B, and 1.35 IU/mL in group C. Statistically significant differences were observed between groups A and C (p = 0.005), but not between groups A and B (p = 0.745) or between groups B and C (p = 0.264). The concentration of hornet venom–specific IgE correlated positively with BAT results for wasp venom (rho = 0.215, p < 0.05).

For Pol d 5, the mean sIgE concentrations were 0.369 IU/mL in group A, 0.361 IU/mL in group B, and 2.80 IU/mL in group C. Statistically significant differences were observed between groups A and C, as well as between groups B and C (p < 0.001 for both), but not between groups A and B (p = 0.853). The concentration of Pol d 5–specific IgE correlated positively with BAT results for wasp venom (rho = 0.342, p < 0.001) and negatively with BAT results for honeybee venom (rho = –0.272, p = 0.008).

### Severity of anaphylactic reactions in patient history

Patients with mild reactions did not differ significantly from those with severe reactions in any of the analyzed parameters, regardless of whether allergen specific IgE concentrations or basophil reactivity were assessed. This observation applied to participants qualified for both wasp venom and honeybee venom immunotherapy (Table 4).

**Table 4:** Measurement results regarding anaphylaxis severity.

Variable	Group of mild reactions			Group of severe reactions			p
	Mean (95% CI^a^)	Standard deviation	Median	Mean (95% CI^a^)	Standard deviation	Median	
Participants qualified for bee venom immunotherapy							
Number of participants	**7**			**25**			N/A
Tryptase [µg/L]	**6.11** **(±1.09)**	1.47	5.9	**6.71** **(±1.01)**	2.58	6.06	0.929
BAT positive control [%]	**59.07** **(±22.74)**	30.70	75.2	**51.28** **(±8.22)**	20.97	51.35	0.365
BAT negative control [%]	**1.71** **(±1.96)**	2.64	0.68	**1.42** **(±0.46)**	1.17	1.3	0.267
BAT honeybee [%]	**34.68** **(± 29.28)**	39.52	8.8	**45.22** **(±13.01)**	33.19	52.7	0.452
sIgE honeybee [IU/mL]	**36.26** **(±30.06)**	40.58	17	**10.34** **(±4.39)**	11.2	6.09	0.314
rApi m 1 [IU/mL]	**15.98** **(±19.22)**	25.94	2.7	**4.67** **(±3.09)**	7.87	1.27	0.869
rApi m 2 [IU/mL]	**3.79** **(±4.22)**	5.69	0	**1.87** **(±1.4)**	3.62	0.15	0.737
rApi m 3 [IU/mL]	**0.44** **(±0,47)**	0.64	0.25	**0.93** **(±1.12)**	2.85	0.07	0.806
rApi m 5 [IU/mL]	**0.47** **(±0.59)**	0.79	0	**0.96** **(±0.4)**	3.14	0.05	0.310
rApi m 10 [IU/mL]	**8.46** **(±6.90)**	9.32	4.67	**1.74** **(±0.98)**	2.51	0.37	0.141
Participants qualified for wasp venom immunotherapy							
Number of participants	**28**			**67**			N/A
Tryptase [µg/L]	**7.76** **(±1.83)**	4.94	5.84	**8.57** **(±1.68)**	7.02	5.7	0.987
BAT positive control [%]	**61.39** **(±7.78)**	21.01	62.04	**62.56** **(±5.51)**	23.02	65.09	0.690
BAT negative control [%]	**2.97** **(±2.64)**	7.12	1.21	**2.71** **(±1.27)**	5.29	1.1	0.894
BAT wasp [%]	**47.70** **(±11.13)**	30.05	52.15	**44.30** **(±6.61)**	27.62	51.4	0.557
sIgE wasp [IU/mL]	**7.96** **(±3.1)**	8.36	5.14	**11.05** **(±4.98)**	20.80	3.49	0.350
rVes v 1 [IU/mL]	**1.78** **(±1.28)**	3.46	0.07	**2.44** **(±2.11)**	8.83	0.09	0.747
rVes v 5 [IU/mL]	**7.33** **(±3.47)**	9.36	2.94	**9.85** **(±1.07)**	4.48	2.33	0.663

^a^Confidence interval.

## Discussion

Our study provides new data for the Central European population, in which few previous reports have examined the concurrent use of BAT and CRD [8,9]. The results indicate that, within our study group, the levels of sIgE to individual recombinant venom components are differentially correlated with basophil reactivity to the corresponding venom solutions. Among the examined variables, Api m 1 and Ves v 5 appear to show the strongest correlation. Interestingly, both of these components are characterized by relatively low molecular weight. Api m 1, a phospholipase A₂ that constitutes approximately 16% of the dry weight of honeybee venom, also exhibits hydrolytic activity leading to pore formation in cell membranes, cell lysis, and the release of additional proinflammatory mediators. Moreover, it exerts non-catalytic, receptor-mediated neurotoxic effects. In contrast, the biological function of Ves v 5, the wasp venom antigen 5, remains unclear [14].

The key roles of Api m 1 and Ves v 5 molecules have already been emphasized in previous studies [15,16]. However, our findings additionally suggest an interesting negative correlation between these components and the results of heterologous basophil activation tests. This phenomenon undoubtedly warrants further investigation, for instance regarding the potential inhibitory effect of antibodies on heterologous FcεRI binding sites, which may lead to reduced basophil activation.

Other venom components appear to be less strongly correlated with basophil responses in our study population. In particular, the Ves v 1 component in our study did not show any correlation with BAT results. This finding is noteworthy in the context of the current recommendations presented in the *EAACI Molecular Allergology User’s Guide 2.0*, which classifies Ves v 1, alongside Ves v 5, as a major wasp venom allergen and considers it an indicator supporting qualification for SCIT. On the other hand, the remaining observed correlations in our study population are fully consistent with the above-mentioned recommendations — similarly to the lack of correlation with Api m 5-specific IgE levels, whose presence does not indicate genuine honeybee venom allergy but rather reflects cross-reactivity [14].

At present, it is difficult to determine the potential clinical relevance of the observed relationships. The study did not demonstrate differences in the analyzed laboratory parameters between patients with mild and severe allergic reactions. However, these analyses were inconclusive, primarily due to the limited size of some subgroups, e.g., the honeybee mild-reaction subgroup. Therefore, the results should be interpreted with caution and should not be used to draw firm conclusions. Further research is needed in this area, preferably in a prospective and multi-center setting – especially to identify potential biomarkers of anaphylaxis severity, such as platelet-activating factor acetylhydrolase (PAF-AH) [17].

Although IgE measurements for Pol d 5 and hornet venom were available only for a subset of patients and therefore should be interpreted with caution, these data provide an interesting perspective on cross-reactivity among *Vespula* and *Polistes* species. This finding suggests a potential diagnostic value of Pol d 5 in regions where *Polistes* exposure is more prevalent, such as Southern Europe. Similarly, the limited data on hornet venom sensitization were consistent with the overall IgE and BAT patterns observed for wasp venom, further supporting the functional and molecular overlap between these *Hymenoptera* species. From a Central European perspective, including Poland, such data are of growing interest due to the expanding geographic range of *Polistes* wasps and *Vespa velutina*, driven by progressive climate change.

As observed during data analysis, a positive BAT result may also occur in the presence of low sIgE levels, a finding that has been attributed to the functional activity of antibodies as well as to high cellular reactivity — a phenomenon previously reported by other authors [10]. Conversely, the absence of basophil activation despite detectable sIgE may indicate a lack of immunological relevance of the sensitization, for example due to the inability of the specific IgE antibodies to trigger basophil activation.

In our opinion, the presented results suggest that combining BAT with CRD may offer complementary insights for the clinical assessment of patients allergic to *Hymenoptera* venom [7,8]. The aforementioned integration may further provide additional information to support decision-making regarding the initiation of immunotherapy, particularly in patients with polysensitization or an inconclusive clinical history in our population.

The main limitations of our study include its retrospective, single-center design and the absence of a healthy control group. The statistical approach applied in this study reflects the exploratory nature of the analysis. Although multiple comparisons were performed, the results should not be interpreted as confirmatory. Rather than relying solely on nominal statistical significance, we emphasize the magnitude of observed effects together with their confidence intervals. Accordingly, the presented findings should be considered hypothesis-generating and require confirmation in larger, adequately powered studies with prespecified primary endpoints.

An additional limitation is that some of the diagnostic measures analyzed, particularly component-resolved diagnostics, contributed to routine clinical decision-making. As a result, between-group comparisons are descriptive in nature and some degree of circularity cannot be excluded (Table 5).

**Table 5:** Classification-related and evaluation-only variables in the context of potential circularity.

Classification-related variables	Evaluation-only variables
Allergic reaction severity	Age and gender
Specific IgE to whole venom	Morphology parameters
Component-resolved diagnostics (Api m 1, m 2, m 3, m 5, m 10, Ves v 1, v 5, Pol d 5)	Tryptase
	Basophil activation test

An important limitation of this study is the absence of skin test data and the use of a single concentration in the BAT protocol. These factors constrain the diagnostic interpretation of the findings. In particular, the lack of skin test results prevents a comprehensive comparison with standard diagnostic modalities, while the use of a single BAT concentration does not allow for assessment of dose–response relationships or determination of optimal diagnostic thresholds. Therefore, the presented results should not be interpreted as providing a comprehensive evaluation of BAT performance. Rather, they reflect associations observed under the specific testing conditions applied in this study. Further studies incorporating a full diagnostic workup, including skin testing and multi-concentration BAT protocols, will be required to more fully characterize the diagnostic utility of BAT.

Several of the above-mentioned limitations were related to resource constraints. For example, BAT was not performed using multiple venom concentrations due to the associated increase in procedural costs, which may have limited the ability to determine an optimal test cut-off. In addition, certain data (e.g., skin test results) were not systematically available in this retrospective registry and were therefore not included in the analysis.

Another limitation concerns missing data. However, the proportion of missing values was low and distributed across study groups without a discernible pattern. Missing data were primarily due to technical or logistical reasons (e.g., insufficient sample material) and were therefore assumed to be missing at random.

## Conclusions

1. Among the examined variables, in our population the major components Api m 1 and Ves v 5 were the most strongly correlated with specific basophil reactivity assessed in vitro in patients allergic to honey bee and wasp venom, respectively.
2. No statistically significant association between the investigated laboratory parameters and the severity of previous anaphylactic reactions was observed in the presented dataset, although these findings should be interpreted as inconclusive given the limited size of some subgroups.
3. Further studies are needed to evaluate the impact of CRD and BAT results on the clinical course of Hymenoptera venom allergy, as well as to identify potential biomarkers of anaphylaxis severity.

## Supporting information

S1 Table Anonymized source data underlying all analyses presented in the manuscript.
(PDF)

S2 Table Complete correlation matrices analyzed in the manuscript.
(PDF)

S3 Table Distribution of missing data in the study dataset.
(PDF)
