# Supplementary material for: Association between results of component-resolved diagnostics and basophil activation in Hymenoptera venom allergy: A registry-based cross-sectional study in adults
Source: PLoS One. 2026 Jun 17;21(6):e0350189. doi: 10.1371/journal.pone.0350189 (PMC13274868; doi:10.1371/journal.pone.0350189)
Supplement: S1 Table — Anonymized source data underlying all analyses presented in the manuscript. (PDF) [file pone.0350189.s001.pdf]

No	Gender	Age	VIT	Tryptase	Api m 1	Api m 2	Api m 3	Api m 5	Api m 10	Ves v 1	Ves v 5	
**1**	2	79	1	12,6	1,27	0,29	0	0	0	0,01	0	
**2**	2	39	1	7,05								
**3**	1	65	2	3,99	0	0	0	0,01	0,01	3,63	11,3	
**4**	1	55	0	3,64	0	0	0	0	0	0,14	0,18	
**5**	1	51	2	5,8						0,14	35,7	
**6**	1	66	2	4,55								
**7**	2	32	0	5,2	0	0	0	0	0	0	0	
**8**	1	54	0	0	0	0,02	0	0	0	0,02	0,09	
**9**	1	35	1	4,92	18,6	0,01	0,08	0,07	0,17	0	0	
**10**	1	45	1	3,4	22,9	11,4	0,29	0	4,67	0,07	0,03	
**11**	1	78	2	5,17						16,5	9,86	
**12**	2	38	0	5,27	0	0	0	0		0,03	0,2	
**13**	1	65	0		0,04	0			0,4	0,09	0,49	
**14**	1	52	1	4,3	0,62	11,4			2,4	0,09	0,1	
**15**	2	35	0		0	0	0	0,25	0	5,65	0,01	
**16**	1	52	1	5,17	6,92	12,9	0,01	0,1	0,05	0,12	0	
**17**	2	34	1	5,69	2,7	2,55	0,23	1,71	0,41	0,87	0,66	
**18**	1	68	2	8,09	0	0	0,02	5,9	0,04	0,09	75,7	
**19**	1	51	2	4,19	0,01	0	0,02	0,01	0,01	2,08	0,02	
**20**	1	71	1	4,88	1,33	0,4	0,04	4,28	7,09	4,73	0,27	
**21**	2	29	2	12,2	0	0	0		0	0	8,01	
**22**	2	66	1	12,5	0,07	0,28	0	0,06	0,14	0	0	
**23**	2	51	2	5,27	0	0	0	0,04	0	0,03	1,83	
**24**	1	55	2	2,96	0	0,02	0,09	0,19	0	0,21	37,8	
**25**	2	45	2	11,5	0	0	0	0	0	0	4,31	
**26**	2	69	2	28	0,02	0,03	0,05	0	0,41	2,03	3,76	
**27**	2	41	2	4,14	0	1,06	0,03	0	0,03	0,03	22,7	
**28**	1	40	0	6,27	0,01	0,01	0	0	0,01	0,04	0,01	
**29**	2	45	2	12,9	0	0	0	0	0	0	4,31	
**30**	1	31	0		0,01	0,01	0,04	0,04	0,02	0,27	0,44	
**31**	1	22	2	2,46	0	0	0,01	0,08	0	8,95	0	

**32**	2	46	2	3,9	0,01	0,25	0,03	0	0	0,06	15,5	
**33**	1	62	2	17	0	4,8	0	0,03	0	0,06	2,01	
**34**	1	65	1	3,78	2,72	0	0,59	0,01	0,58	0,19	0	
**35**	2	53	0	3,56	0,7	0	0	0	0,33	0	0,08	
**36**	1	43	2	3,29	0	0	0,01	0	0	68,7	26,9	
**37**	2	36	0	4,14	0,01	0	0,01	0	0	0,01	0	
**38**	1	44	0	3,86	0,42	0,67	0,03	0,02	0,05	0,01	0,13	
**39**	2	54	1	5,72	0,82	0	0,01	0,02	0	0,11	0	
**40**	2	37	2	8,88	0,06	0,03	0,08	1,2	0,07	1,25	12,6	
**41**	1	69	1	5,9	15	12,6	0,25	0	19,8	0,01	0	
**42**	2	41	2	27	0	0	0	0	0	0,62	0,51	
**43**	2	49	2	21,2	0,01	2,98	0,06	0,02	0	0,79	0,86	
**44**	1	58	1	4,94	0,45	0,96	0,05	0,22	0,05	0,39	0,83	
**45**	1	60	0	8,39	0	0	0	0	0	1,42	0,61	
**46**	1	52	2	13,8	0	0	0	0,09	0,03	0,51	0,24	
**47**	2	45	2	23,7	0	0	0	0	0	0,04	0,14	
**48**	1	65	2	5,1	0,01	0,02	0,02	28,6	0	2,61	15,1	
**49**	1	49	2	2,57	0,1	0	0,17	6,78	0,04	0,01	0,77	
**50**	1	75	2	13,9	0	0	0	0	0	6,16	15,9	
**51**	2	32	1	7,49	0,03	0	1,85	0	13,7	0	0,08	
**52**	2	53	2	9,15	0	0	0	0	0	0,06	0,06	
**53**	1	52	2	5,01	0,01	0,01	0,07	0,01	0,01	2,42	10,1	
**54**	2	34	1	10,3	0	0,01	0,01	0,03	0,13	0,93	0,71	
**55**	2	71	0	15,4	0	0,01	0	0	0	0,01	0	
**56**	1	39	2	5,07	0	0,01	0,02	0	0	0,01	2,25	
**57**	1	56	0	3,65	0,04	0,02	0,01	3,22	0,04	1,17	0,58	
**58**	2	56	2	4,89	0	0,02	0,01	0,47	0	0,74	0,47	
**59**	1	47	2	17,9	0	0	0	0	0	0,02	1,32	
**60**	2	46	2	12,1	0	0	0,02	0	0	4	3,45	
**61**	2	65	2	5,99	0	0	0	0	0	0	7,47	
**62**	1	46	2	11,3		0	0	0	0	0	16,8	
**63**	1	38	2	7,64	0	0	0	0	0	1,76	0,16	
**64**	1	48	2	5,71	0,01	0	0	0	0	0,67	2,16	

**65**	2	59	2	3,79	0	0	0	0	0	0,01	9,69	
**66**	1	40	2	6,14	0,01	0,01	0,55	0,29	0,01	0,01	2,69	
**67**	1	47	2	4,77	0	0	0	0	0	0	0,62	
**68**	1	53	1	6,53	0,09	0,61	0,01	0	0,01	0,25	0	
**69**	1	45	2	3,74	0	0	0	0	0	0	0,22	
**70**	1	23	1	11,5	1,26	6,55	1,04	0,16	4,04		0,01	
**71**	1	74	2	5,06	0	0	0	0	0	0,01	3,46	
**72**	1	50	2	17,8	0	0,1	0	0	0	1,05	0,68	
**73**	2	47	2	14,5	0	0,01	0	0,02	0	4,14	0,5	
**74**	1	46	2	35	0	0	0	0	0	0	0,39	
**75**	2	50	2	12,3	0,02	0,02	0,01	0,01	0,01	0,01	16,9	
**76**	1	45	0	4,42	0,54	0,01	0,16	0,01	1,13	0,02	0,02	
**77**	1	49	2	10,3	0,01	0,01	0,04	7,27	0,02	0,03	6,45	
**78**	2	21	0	6,53	0,26	1,44	0,34	0,43	1,74	0,01	0,13	
**79**	1	69	2	6,83	0,02	0,02	0,01	0,01	1,23	1,75	3,02	
**80**	2	75	2	5,71	0,06	0,02	0,02	0,03	0	0,01	0,19	
**81**	1	65	2	8,82	0,01		0,01		0,01	0,01	9,63	
**82**	2	74	2	6,7	0	0	0	0,01	0,01		5,4	
**83**	2	55	1	6,98	33,8	0,2	2,01	0,02	1,57	0,01	0,05	
**84**	2	71	2	6,24	0,05	0,01	0	1,8	0,35	6,91	2,06	
**85**	2	27	1	6,06	5,17	0,02	2,18	14,7	7,28	0,04	0,12	
**86**	1	43	2	12,8	0	0	0	0,01	0	0,05	1,76	
**87**	2	57	2	7,56	0	0	0	0	0	0,21	0,12	
**88**	2	64	2	6,01	0,03	0,62	0,01	0,01	0,05	3,71	8,41	
**89**	2	57	2	8,34	1,11	0,89	0,32	19,8	0,06	16	0,14	
**90**	1	57	2	5,7	0,01	0	0	0	0	8,31	1,41	
**91**	2	38	2	3,6	0	0	0	0	0	0	5,61	
**92**	1	37	1	6,46	0,7	0	0,14	0	0,06	0	0	
**93**	1	23	0	2,61	0,01	0	0	0	0	0,09	0,78	
**94**	2	49	2	11,2	0	0,01	0	0,11	0	4,27	2,34	
**95**	2	36	2	3,51	0	0	0,06	0	0	0,04	1,55	
**96**	2	38	2	11,8	0	0	0	0	0	0,66	0	
**97**	2	45	2	23	0	0	0	0	0	0,43	0,14	

**98**	1	47	2	4	0	0	0,02	0	0	0	0,59	
**99**	1	27	2	2,61	0	0	0	0	0	0,07	17	
**100**	2	45	1	8,09	13,8	0	0,07	0,02	0,02	0,08	0,4	
**101**	1	50	0	5,19	0	0			0	0,02	0,17	
**102**	2	29	1	4,93	10,9	0,03	0,75	0,4	1,93	0,04	0,13	
**103**	1	68	0	19,9	0	0	0	0	0	0	0	
**104**	2	35	2	3,32	0	0	0	0	0	0	0,93	
**105**	2	57	2	4,05	0,1	0,04	0,07	0,05	0,06	0,05	30,5	
**106**	1	72	2	4,85	0,26	0	0,14	0,32	0,28	0,04	1,07	
**107**	1	66	2	4,85	0,01	0,01	0,01	0,01	0,01	0,22	0,96	
**108**	1	79	0	10,1		0	0	0	0	0,07	0,13	
**109**	1	51	2	4,79	0,01	0	0,11	0,11	0,01	0,06	2,94	
**110**	1	39	2	3,45								
**111**	2	39	0	4,32	0	0	0,02	0,06	0	0	0,07	
**112**	1	38	1	5,03	0,26	0	0,08	0,05	3,76	0,01	0,05	
**113**	2	40	2	4,19	0	0	0,03	0	0	0,02	2,89	
**114**	1	53	2	3,3	0,01	0,02	0,05	0		0,13	0,95	
**115**	1	52	2	8,72	0	0	0,04	0,03	0,01	0,04	1,94	
**116**	1	40	2	4,49	0	0	0,02	0,02	0,2	1,21	4,86	
**117**	2	37	2	10,9	0	0	0	0	0	0,01	1,8	
**118**	1	45	1	6,01	3,17	0,04	13,7	0,05	1,84	8,39	8,54	
**119**	2	38	2	17,1	0	0	0,01	0,07	0	0,2	0,44	
**120**	1	67	0	2,63	0	0	0	0	0	0,01	0,19	
**121**	1	48	2	4,31	0,01	0	0,01	0,15	0,04	0,04	6,05	
**122**	2	38	2	5,36	0	0	0,01	0	0,01	0,02	1,16	
**123**	1	60	1	5,83	71,2	0	0,43	1,52	20,5	0,01	0,09	
**124**	1	37	2	4,65	0	0	0	0,04	0	0,03	4,93	
**125**	1	73	0	9,13	0	0	0	0,04	0	0,16	0	
**126**	1	49	0	5,28	0	0,01	0	0	0	0,23	4,04	
**127**	2	46	2	7,62	0	0,02	0	0	0	0,09	1,58	
**128**	2	46	1	6,72	6,51	4,78	0,32	1,67		0,03	0,01	
**129**	1	51	2	3,5	0	0	0	0	0	0	0,71	
**130**	1	53	2	6,44	0	0	0	0	0	0,67	2,07	

**131**	1	56	0	3,56	0,03	0	0,03	0	0	0,01	0	
**132**	2	24	2	2,44	0	0	0	0,05	0	4,8	37,7	
**133**	1	67	2	5,84	0	0	0	0,11	0	0,07	9,4	
**134**	1	40	2		0	0	0	0,01	0	0,11	22,3	
**135**	1	40	2	5,94	0	0,02	0	0	0	3,86	0,62	
**136**	1	68	1	4,55	0	0	0,27	0	7,62	0,01	0,08	
**137**	2	42	2	4,22	0	0	0,03	0,05	0,19	4,74	0,93	
**138**	2	39	1	7,92	0	0	0	0	0	0,33	0,12	
**139**	1	39	2	3,32		2,98	0,01	0	0	0,01	4,61	
**140**	1	76	1	6,51	0,04	0	0	0,03	0,15	0,12	0,01	
**141**	2	51	2	3,23	0,28	0,31	0,45	0,39	0,68	2,67	24,8	
**142**	1	68	0	6,7	0	0	0,08	0	0	0	0,11	
**143**	1	49	0	3,1	0,23	0	0,01	0	0,02	0	0,32	
**144**	2	35	1	7,13	3,08	4	0,02	0,01	0,37	0	0,02	
**145**	1	64	2	25,3	0	0	0	0	0	0,22	0,42	
**146**	2	42	2	4,24	0	0	0	0	0	0	2,31	
**147**	1	50	2	2,57	0	0	0	0	2,13	0	5,36	
**148**	1	63	2	15	0,01	0	0	0	0	7,34	0,53	
**149**	2	52	2	4,17	16,7	0,3	1,97	0,76	1,66	7,3	12,7	
**150**	2	72	2	4,19	0,06	0,01	0,02	0	0,21	0,05	100	
**151**	1	19	1	3,24	0,35	0,1	0,06	0,08	0,64	0,03	0,01	
**152**	1	40	2	3,69	0,01	0,01	0,01	0,64	0,02	1	9,54	
**153**	1	54	1	8,24	0,18	2,34	0	0,02	0,23	0,12	0,04	
**154**	2	49	2	9,68	0,02	0,02	0,02	0,02	0,03	0,08	100	
**No**	**Pol d 5**	**Honeybee**	**Wasp**	**Hornet**	**NEUT**	**EOS**	**BASO**	**WBC**	**BAT neg**	**BAT pos**	**BAT honeybee**	**BAT wasp**
**1**		2,57	0,07	0,16	3,24	0,21	0,01	6,06		53,1	0	54,7
**2**		10,5			4,1	0,01	0,03	6,01	1,46	45,47	3,23	85,47
**3**		0,46	24,8	2,18	3,05	0,08	0,05	6,61			20	0
**4**					2,67	0,11	0,05	6,27		20,8	2,4	1,3
**5**		0,37			1,45	0,15	0,03	5,21			55,3	5,7
**6**		0,01	2,1		3,86	0,08	0,04	6,98			92,9	4,1
**7**		0,02	0,01	0,04	3,12	0,05	0,03	6,67		23,4	3,2	3,2

**8**		0	0	0,03	3,7	0,14	0,01	6		26,1	13,4	7
**9**			0,16		3,4	0,14	0,03	5,44			4,2	86,8
**10**		40,2	0,18		3,78	0,13	0,03	6,62		75,2	2,8	85,2
**11**		0	26,1		1,65	0,08	0,06	4,5		23,7	5,8	3
**12**		0,1	0,69	1,7	3,6	0,18	0,05	6,4		26,9	4,6	21,5
**13**		0,51	0,67		3,42	0,08	0,03	6,57		84,9	4,2	0,3
**14**		31,2	0,56		2,19	0,1	0,03	4,14		19,8	2,5	62,9
**15**		0,24			1,82	0,16	0,03	3,76	0,4	85,1	16	0,8
**16**					3,63	0,23	0,03	7,53	1,3	77,6	0,9	78,7
**17**		17	4,07		2,51	0,25	0,06	5,48	0,2	84,9	43,9	81,8
**18**					3,93	0,18	0,05	6,57	0,7	31,9	9,8	1,9
**19**		0	2,1		4,56	0,19	0,06	6,58	0,2	54,1	49,7	0,6
**20**		29,3	7,86	7,1	2,34	0,07	0,03	4,07		55,8	0	74,6
**21**					2,77	0,12	0,04	5,45	2,6	42,4	5,5	1,2
**22**		2,1			2,33	0,1	0,02	3,91	1,4	83,6	3,3	83
**23**		0,01	2,02	0,51	4,91	0,2	0,05	8,12	1,79	79,6	85,1	1,9
**24**	25,3	0	14,1	2,25	2,19	0,07	0,03	4,42	2,51	40,9	57,1	1
**25**		0	5,14		1,76	0,09	0,03	3,78	0,21	78,69	91,3	0
**26**	2,25	2,75	5,31	1,79	3,12	0,17	0,07	5,5	0,54	89,8	81,3	6,1
**27**		0,96	23	0,59	5,35	0,12	0,02	7,68	3,9	66,3	77,5	4,3
**28**	0	0,02	0,05	0,06	1,27	0,08	0,01	3,41	2,58	88,24	25,5	3,5
**29**		0	5,14	6,17	2,35	0,12	0,04	4,51	0,21	78,69	91,3	0
**30**			0,51	0,15	2,81	0,19	0,06	5,2	1,68	87,82	11,3	1,9
**31**	0,07	1,32	9,16	1,74	7,56	0,18	0,02	10,59	0,33	92,3	85,8	8,5
**32**	12,3	0,03	13,2	0,11	2,29	0,2	0,05	4,312	1,5	35,3	40	1
**33**	0,56	3,7	2,95	1,03	2,42	0,21	0,04	4,53	1,2	59,5	66,3	0
**34**	0	4,03	0,18	0,05	2,55	0,12	0,05	4,55	0,1	26,01	0,2	44,8
**35**	0,02	0,33	0,07	0,06	2,51	0,06	0,05	4,97	2,9	57,62	1,4	9
**36**	2,59	0,13	87,3		5,8	0,05	0,01	8,12	4,42	17,17	19,6	2,8
**37**	0	0,01	0,01		4,12	0,19	0,04	7,68	10,6	80,2	12	5,8
**38**	0	1,22	0,18		4,76	0,08	0,03	7,16	9,6	31,8	8,7	11,7
**39**	0	0,79	0		2,84	0,35	0,04	5,44	0,8	85,3	66,7	86,5
**40**	7,63	1,48	10,7	1,52	2,54	0,15	0,09	5,89	4,9	52,8	57,5	4,9

**41**	0	100	0,12	0,56	6,69	0,1	0,02	9,57	7,07	33,7	0	61,2
**42**	0,21	0,01	1,14	0,09	2,69	0,12	0,02	4,98	3,5	38,4	74,8	4,6
**43**	0,54	5,05	3,12	2,16	2,4	0,15	0,05	4,95	1,6	88,3	35,5	47,8
**44**	0,41	10,5	10,5		5,17	0,07	0,02	7,66	0,83	17,14	4	36,4
**45**	0,23	0	2,03	0,09	3,78	0,26	0,03	7,59	4,95	28,72	1	1,5
**46**	0,21	0,31	1,06	0,34	3,97	0,19	0,03	7,59	1,4	80,27	54,9	3
**47**	0,08	0,08	1,2	0,55	4,42	0,02	0,02	6,75	13,67	46,24	5,2	10,6
**48**	1,55	1,35	19,4	13,6	6,83	0,09	0,06	9,53	1,2	73,2	60,1	21
**49**	0,18	1,1	1,44	4,19	3,16	0,05	0,04	4,96	2,29	64,07	31,3	11,1
**50**	7,05	0,01	21,4	1	4,98	0,34	0,1	7,35	3,53	39,23	5,5	39,2
**51**		12,5	0	0	3,04	0,19	0,03	6,87	0,6	46,6	0,6	3,5
**52**	0,02	0	0,15		2,19	0,09	0,03	5,38	3,6	66,1	45,5	6,6
**53**		0,03			3,58	0,02	0,05	6,27	0,16	55,17	78,7	0,1
**54**	0,45	1,43	2,25	1,23	2,94	0,35	0,04	6,4	1	44,2	13,6	1,2
**55**	0	0,01	0,01	0,04	3,32	0,06	0,01	5,25	4,4	57,5	6,4	3,7
**56**	0,11	0			4,09	0,11	0,05	7,4	1,08	23	3	1,5
**57**		2,18	2,72	1,74	2,22	0,15	0,03	4,77	0,3	25,7	19,5	1,3
**58**	0,03	0,07	1,17	0,14	2,18	0,14	0,06	4,5	0,2	58,7	54,7	0
**59**	0,14	0	2,1	0,15	3,25	0,18	0,04	6,97	2,94	52,6	77,1	0,5
**60**		0	7,19	0,18	5,01	0,01	0,04	6,88	0,54	12,65	41,4	0,1
**61**	0,11	0	13,43	0,09	2,2	0,1	0,04	5,43	1,7	37,5	75,6	1,4
**62**	2,13	0,03	30,9	0,2	5,69	0,11	0,04	7,83	0,37	78,37	79	0,4
**63**		0,1	5,31	0,09	3,97	0,24	0,03	5,7	4,74	84,44	54,6	4,4
**64**		0,24	2,56	0,24	4,07	0,02	0,08	6,53	1,11	90,6	83,3	2,2
**65**	6,34	0,01	7,44		2,93	0,04	0,06	5,03	1,15	90,95	45,4	0,3
**66**	0,42	0,14	2,23	0,05	3,67	0,17	0,04	6,16	0,16	68,9	68,9	0
**67**	0,24	0	1,04	0,05	4,16	0,07	0,03	6,47	1,64	44,56	32	2,2
**68**	0	0,9	0,27	0,07	2,4	0,05	0,06	4,69	1,2	52,7	3,5	52,7
**69**	0,05	0,09	1,02	0,05	2,53	0,18	0,06	5,16	0,5	47,8	4,9	0
**70**	0,02	17,5	0	0	5,44	0,05	0,06	7,19	1,6	53,9	0	54,9
**71**	1,09	0,01	4	0,12	2,46	0,14	0,02	5,04	1,3	47	54,6	0
**72**	0,25	0,02	1,69	0,18	3	0,06	0,02	5,24	36,75	91,44	65,6	15,6
**73**	0,58	0,02	3,49	0,06	3,56	0,1	0,02	5,84	1,89	38,88	3,6	2

**74**	0,11	0,01	0,44	0,04	2,92	0,09	0,03	4,99	0	42,2	31,2	1,5
**75**	1,02	0,09	16,6		3,82	0,04	0,04	6,47	0,96	43,5	4,3	0,3
**76**	0,03	6,12	0,22	0,52	3,44	0,15	0,04	6,56	3,5	62,7	4	35,9
**77**	9,16	0,94	8,26	4,73	2,75	0,07	0,03	4,62	6,1	67,4	66,3	2,1
**78**	0,94	6,74	0,12	0,23	2,66	0,09	0,02	4,3	2,91	49,83	1,7	13,8
**79**	1,17	1,37	13,5	0,56	6,47	0,07	0,05	9,75	1,6	87,8	51,4	1,3
**80**	0,05	0,15	0,2	0,07	3,51	0,15	0,04	4,96	0,3	90,2	53	15,6
**81**		0,13	6,35	0,36	3,63	0,49	0,06	6,05	0,8	37,5	1,5	1
**82**	2,56		7,6	9,79	3,45	0,05	0,04	6,11	0	32,74	5,4	0
**83**	0,14	41,7	1,72	4,19	3,3	0,29	0,07	7,11	1,5	42,5	1,8	24,6
**84**	0,52	0,45	8,53	2,82	4,61	0,2	0,04	7,16	14	35,2	32,7	13
**85**		13,9	0,72	1,59	2,85	0,02	0,03	4,12	1,2	73	3	72,5
**86**	1,89	0,02	2,44	0,69	3,41	0,26	0,08	7,79	1,1	37,9	5,3	4,4
**87**	0,07	0,02	0,4	0,17	3,92	0,07	0,03	7,24	8,79	80,03	5,1	4,4
**88**	2,36	0	3,5	3,5	2,95	0,31	0,05	6,29	3,8	71,3	62,1	4,4
**89**		2,47	10,2	0,47	2,03	0,22	0,03	4,3	6,7	84,3	60,7	86,6
**90**	0,06	0,02	8,31		4,32	0,29	0,07	7,75	0,6	74,2	51,6	0,6
**91**	4,39	0,09	5,03		4,05	0,1	0,07	7,71	1,4	57,3	25,1	1,3
**92**		0	0		3,82	0,11	0,04	6,26	0,8	58,7	2	15,5
**93**	0,03	0,03	0,53		5,81	0,16	0,06	7,96	1,4	69,9	7,6	2,1
**94**	2,35	0	4,93	0	5,57	0,17	0,04	9,98	0,3	87,2	17,9	0,5
**95**	0,14	0,04	1,71	0,24	3,73	0,1	0,04	7,03	0,53	68,5	27,8	3,2
**96**	0	0,01	0,54	0,02	3,03	0,07	0,02	5,4	0,1	85,2	13,7	0,6
**97**	0,15	2,57	0,99	0,78	4,36	0,28	0,02	6,83	0,63	80,9	61,5	16,5
**98**	0,06	0,07	0,56	0,03	2,64	0,11	0,06	5,33	1,4	89,2	50,7	1,5
**99**	9,32	0,07	19,1	0,35	3,23	0,08	0,04	5,63	0,4	42,3	52,4	0,6
**100**	0,3	2,52	0,51	0,06	5,06	0,25	0,03	8,81	0,5	76,3	20	68
**101**		0,02	0,3	0,05	5,28	0,5	0,04	9,15	0,26	90,96	10,2	1,1
**102**		12,9	0,17	0,17	3,81	0,43	0,07	6,72	4,29	72,96	18,8	47,7
**103**	0	0	0,01	0,02	4,11	0,15	0,06	6,97	2,68	23,28	2,7	4,6
**104**	3,46	0	0	0	3,15	0,12	0,06	5,61	0	94,9	72,7	0
**105**		0,22	31,3	0,66	4,57	0,16	0,06	8,01	0,8	59,8	41,9	0
**106**		3,5	0	0	2,04	0,25	0,01	5	2	82,8	59	54,5

**107**	0,97	0,02	0,86		2,88	0,05	0,04	6,07	0,2	43,6	0,2	0,6
**108**		0,01	0,14	0,05	3,24	0,07	0,01	5,17		18	6,6	8,5
**109**		0,05			1,91	0,19	0,03	4,2	38,1	84,9	78,5	54,2
**110**		0,01	7,33	0,7	2,67	0,09	0,02	4,47	0,5	40	81,7	0,1
**111**	0,02	0,01	0,18	0,19	4,31	0,03	0,03	7,12	2,2	78,3	1,3	2
**112**		3,43	0,03		3,79	0,04	0,08	6,83	1,4	29,7	2,2	1,8
**113**		0,02	3,49		4,78	0,1	0,06	9,4	6,83	75,09	82,9	28,9
**114**		0,48	2,07	0,36	4,22	0,12	0,04	7,21	11	43,2	51,8	12,5
**115**		0	0		5	0,12	0,06	9,05	0,6	21,55	1,7	0,6
**116**		0,19	5,44		6,39	0,23	0,08	9,42	1,7	58,6	82,9	1,2
**117**	0,97	0,1	1,71		3,54	0,14	0,02	6,36	1,3	92	89,1	1,3
**118**	4,08	17,5	17,5	0,7	3,37	0,07	0,03	6,12	1,68	27,7	1,2	2,1
**119**	0,39	0,01	0,93	0,18	3,98	0,1	0,01	6,53	1,1	83,3	39,9	2,7
**120**	0,25	0,02	0,19		1,85	0,4	0,05	3,72	1,15	74,87	8,3	0,2
**121**	2,62	0	1,05	0,06	2,05	0,2	0,06	5,05	0,78	88,6	40,7	0,2
**122**		0,01	2,23	0,42	3,64	0,13	0,02	6,72	0,57	65,38	34,2	1
**123**		83,8	0,14	0,07	3,79	0,05	0,8	6,28	0,77	6,79	0,6	1,2
**124**	0,6	0	3,5		4,78	0,03	0,03	7,4	2,8	71,9	71,9	2,8
**125**	0	0,02	0,2	0,24	1,92	0,06	0,05	3,65	1,8	81,7	50,3	3,2
**126**	4,2	0	0	0	4,93	0,06	0,02	6,96	1,4	3,7	2,2	56,7
**127**	0,16	0,02	1,84		4,7	0,27	0,03	8,19	1,03	56,7	75,5	1,8
**128**	0,01	17,5	0	0	4,32	0,29	0,03	6,49	1,64	76,89	2,4	86,5
**129**		0,02	1,83	0,08	3,5	0,06	0,03	5,98	2,89	94,7	81,7	0
**130**	1,87	0	4,89	0,05	3,37	0,06	0,04	5,38	0,12	66,7	70,3	0,1
**131**	0	0,01	0,04	0,05	4,72	0,18	0,02	8,04	1,2	1	1,7	6,4
**132**		0,02	34,3	0,6	2,25	0,17	0,03	4,55	0	31,07	3	1,3
**133**	2,85	0,01	8,94	0,64	2,75	0,3	0,07	5,16	1,49	36,59	14,4	0,9
**134**	11,2	0	0,35		4,99	0,14	0,05	7,1	4,9	88,9	57,1	2,6
**135**			3,77		3,76	0,03	0,04	5,21	2,7	70,9	7,4	3,7
**136**	0	7,56	0,1	0,04	6,19	0,05	0,07	8,93	4,4	50	2,8	1,3
**137**	0,11	0,58	6,49	0,25	5,13	0,08	0,06	7,99	1,9	56,8	19,8	2,2
**138**	0	0	0,47	0,03	6,81	0,35	0,1	9,26	0,6	87,4	23,6	1
**139**	3,94	0,01	2,18	0,05	3,73	0,21	0,05	7,94	5,7	93,1	29,9	2,9

**140**	0	0,34	0,15	0,05	3,57	0,26	0,04	6,23	1	78,9	1,5	8,8
**141**		10,4	26,6	9,65	2,38	0,28	0,03	12,9	0	87,9	51,9	0,6
**142**	0,12	0,24	0,15	0,07	6,24	0,19	0,04	10,78	0,9	32	0	0,6
**143**	0,44	1,2	0,1	0,13	4,57	0,1	0,06	7,05	0,3	54,3	0,7	31,3
**144**		6,09	0,13	0,63	4,25	0,2	0,04	8,38	2,8	25,1	0,9	5,4
**145**	0	0	0,71	0,05	5,31	0,02	0,08	15	0,5	51,9	7,2	0,9
**146**	1,29	0,01	2,38	0,28	5,73	0,14	0,06	8,12	0,53	62,6	52,1	0,2
**147**	0,6	0	18,1	0,24	1,82	0,2	0,03	4,57	0,7	88,4	55,4	2,7
**148**	0,01	0	7,02	3,75	3,28	0,15	0,3	6,04	0,7	26,6	20,6	0,6
**149**	5,44	20,8	22	0,99	2,83	0,08	0,04	5,28	0,4	48,9	25,3	25,9
**150**	32,8	0,35	100	2,88	1,62	0,02	0,01	3,58	0,1	73,6	23,5	0,1
**151**	0	1,71			3,62	0,15	0,04	5,21	0	46,8	0,5	1,4
**152**		0,08	16,9	3,28	4,49	0,14	0,04	7,36	0	88,4	71,2	0
**153**		2,19	0,28	0,83	3,92	0,13	0,02	6,64	0	36,5	11,7	1,1
**154**		0,03	100	3,5	5,66	0,02	0,03	7,57	0,41	36,7	1,5	0,5

Variables are coded as follows:

- Gender: 1 = female, 2 = male.
- VIT: 0 = no qualification for venom immunotherapy, 1 = qualified for honeybee venom immunotherapy, 2 = qualified for wasp venom immunotherapy.
