# Supplementary material for: Association between results of component-resolved diagnostics and basophil activation in Hymenoptera venom allergy: A registry-based cross-sectional study in adults
Source: PLoS One. 2026 Jun 17;21(6):e0350189. doi: 10.1371/journal.pone.0350189 (PMC13274868; doi:10.1371/journal.pone.0350189)
Supplement: S2 Table — Complete correlation matrices analyzed in the manuscript. (PDF) [file pone.0350189.s002.pdf]

| | | Age | TRYP | m1 | m2 | m3 | m5 | m10 | v1 | v5 | d5 | Honeybee | Wasp | Hornet | NEUT |
|---|---|---|---|---|---|---|---|---|---|---|---|---|---|---|---|
| Age | rho Spearman | — | | | | | | | | | | | | | |
| | df | — | | | | | | | | | | | | | |
| | p | — | | | | | | | | | | | | | |
| | N | — | | | | | | | | | | | | | |
| TRYP | rho Spearman | 0.130 | — | | | | | | | | | | | | |
| | df | 148 | — | | | | | | | | | | | | |
| | p | 0.112 | — | | | | | | | | | | | | |
| | N | 150 | — | | | | | | | | | | | | |
| m1 | rho Spearman | 0.084 | -0.112 | — | | | | | | | | | | | |
| | df | 144 | 140 | — | | | | | | | | | | | |
| | p | 0.315 | 0.186 | — | | | | | | | | | | | |
| | N | 146 | 142 | — | | | | | | | | | | | |
| m2 | rho Spearman | 0.046 | 0.024 | 0.570*** | — | | | | | | | | | | |
| | df | 146 | 142 | 143 | — | | | | | | | | | | |
| | p | 0.576 | 0.771 | < .001 | — | | | | | | | | | | |
| | N | 148 | 144 | 145 | — | | | | | | | | | | |
| m3 | rho Spearman | -0.112 | -0.227** | 0.657*** | 0.440*** | — | | | | | | | | | |
| | df | 144 | 141 | 141 | 143 | — | | | | | | | | | |
| | p | 0.179 | 0.006 | < .001 | < .001 | — | | | | | | | | | |
| | N | 146 | 143 | 143 | 145 | — | | | | | | | | | |
| m5 | rho Spearman | -0.009 | -0.038 | 0.435*** | 0.362*** | 0.480*** | — | | | | | | | | |
| | df | 142 | 139 | 139 | 142 | 142 | — | | | | | | | | |
| | p | 0.914 | 0.657 | < .001 | < .001 | < .001 | — | | | | | | | | |
| | N | 144 | 141 | 141 | 144 | 144 | — | | | | | | | | |
| m10 | rho Spearman | 0.040 | -0.138 | 0.741*** | 0.443*** | 0.670*** | 0.478*** | — | | | | | | | |
| | df | 144 | 140 | 141 | 143 | 141 | 139 | — | | | | | | | |
| | p | 0.634 | 0.103 | < .001 | < .001 | < .001 | < .001 | — | | | | | | | |
| | N | 146 | 142 | 143 | 145 | 143 | 141 | — | | | | | | | |
| v1 | rho Spearman | 0.138 | 0.091 | 0.012 | 0.183* | 0.021 | 0.304*** | 0.044 | — | | | | | | |
| | df | 147 | 143 | 142 | 144 | 142 | 140 | 142 | — | | | | | | |
| | p | 0.093 | 0.275 | 0.888 | 0.027 | 0.802 | < .001 | 0.604 | — | | | | | | |
| | N | 149 | 145 | 144 | 146 | 144 | 142 | 144 | — | | | | | | |
| v5 | rho Spearman | 0.053 | -0.110 | -0.286*** | -0.033 | -0.058 | 0.059 | -0.129 | 0.217** | — | | | | | |
| | df | 149 | 145 | 144 | 146 | 144 | 142 | 144 | 147 | — | | | | | |
| | p | 0.519 | 0.186 | < .001 | 0.694 | 0.488 | 0.483 | 0.121 | 0.008 | — | | | | | |
| | N | 151 | 147 | 146 | 148 | 146 | 144 | 146 | 149 | — | | | | | |
| d5 | rho Spearman | -0.042 | -0.090 | -0.133 | 0.133 | -0.027 | 0.143 | -0.014 | 0.166 | 0.885*** | — | | | | |
| | df | 93 | 92 | 91 | 93 | 93 | 93 | 92 | 91 | 93 | — | | | | |
| | p | 0.684 | 0.388 | 0.204 | 0.197 | 0.796 | 0.165 | 0.894 | 0.112 | < .001 | — | | | | |
| | N | 95 | 94 | 93 | 95 | 95 | 95 | 94 | 93 | 95 | — | | | | |
| Honeybee | rho Spearman | -0.021 | -0.061 | 0.696*** | 0.517*** | 0.596*** | 0.454*** | 0.685*** | 0.102 | -0.222** | -0.088 | — | | | |
| | df | 144 | 141 | 136 | 138 | 136 | 135 | 136 | 140 | 141 | 92 | — | | | |
| | p | 0.801 | 0.470 | < .001 | < .001 | < .001 | < .001 | < .001 | 0.228 | 0.008 | 0.397 | — | | | |
| | N | 146 | 143 | 138 | 140 | 138 | 137 | 138 | 142 | 143 | 94 | — | | | |
| Wasp | rho Spearman | 0.082 | -0.085 | -0.173* | 0.104 | -0.062 | 0.156 | -0.051 | 0.469*** | 0.792*** | 0.667*** | -0.038 | — | | |
| | df | 140 | 137 | 134 | 136 | 134 | 133 | 134 | 136 | 138 | 91 | 136 | — | | |
| | p | 0.330 | 0.318 | 0.044 | 0.227 | 0.473 | 0.070 | 0.555 | < .001 | < .001 | < .001 | 0.654 | — | | |
| | N | 142 | 139 | 136 | 138 | 136 | 135 | 136 | 138 | 140 | 93 | 138 | — | | |
| Hornet | rho Spearman | 0.082 | -0.033 | 0.184 | 0.278** | 0.157 | 0.366*** | 0.263** | 0.384*** | 0.444*** | 0.423*** | 0.293** | 0.665*** | — | |
| | df | 108 | 107 | 104 | 106 | 106 | 105 | 104 | 105 | 107 | 74 | 106 | 108 | — | |
| | p | 0.396 | 0.736 | 0.059 | 0.004 | 0.104 | < .001 | 0.006 | < .001 | < .001 | < .001 | 0.002 | < .001 | — | |
| | N | 110 | 109 | 106 | 108 | 108 | 107 | 106 | 107 | 109 | 76 | 108 | 110 | — | |
| NEUT | rho Spearman | -0.131 | 0.033 | -0.088 | -0.091 | 0.044 | -0.115 | -0.073 | 0.020 | -0.020 | -0.113 | -0.020 | -0.054 | -0.112 | — |
| | df | 151 | 147 | 143 | 145 | 143 | 141 | 143 | 146 | 148 | 92 | 143 | 140 | 108 | — |
| | p | 0.106 | 0.691 | 0.292 | 0.274 | 0.599 | 0.171 | 0.382 | 0.808 | 0.806 | 0.280 | 0.810 | 0.523 | 0.242 | — |
| | N | 153 | 149 | 145 | 147 | 145 | 143 | 145 | 148 | 150 | 94 | 145 | 142 | 110 | — |
| EOS | rho Spearman | -0.006 | 0.030 | 0.070 | 0.036 | 0.010 | 0.122 | -0.010 | 0.062 | -0.056 | -0.019 | 0.041 | -0.036 | 0.023 | -0.030 |

| | | | | | | | | | | | | | | | |
|---|---|---|---|---|---|---|---|---|---|---|---|---|---|---|---|
| | df | 151 | 147 | 143 | 145 | 143 | 141 | 143 | 146 | 148 | 92 | 143 | 140 | 108 | 151 |
| | p | 0.937 | 0.713 | 0.405 | 0.663 | 0.907 | 0.146 | 0.907 | 0.455 | 0.494 | 0.858 | 0.627 | 0.673 | 0.809 | 0.712 |
| | N | 153 | 149 | 145 | 147 | 145 | 143 | 145 | 148 | 150 | 94 | 145 | 142 | 110 | 153 |
| BASO | rho Spearmana | 0.042 | -0.076 | 0.001 | -0.152 | 0.086 | 0.039 | 0.035 | 0.041 | 0.176* | 0.034 | -0.044 | 0.088 | 0.065 | 0.134 |
| | df | 151 | 147 | 143 | 145 | 143 | 141 | 143 | 146 | 148 | 92 | 143 | 140 | 108 | 151 |
| | p | 0.607 | 0.358 | 0.993 | 0.065 | 0.305 | 0.647 | 0.676 | 0.621 | 0.031 | 0.742 | 0.601 | 0.298 | 0.498 | 0.100 |
| | N | 153 | 149 | 145 | 147 | 145 | 143 | 145 | 148 | 150 | 94 | 145 | 142 | 110 | 153 |
| WBC | rho Spearmana | -0.118 | -0.021 | -0.086 | -0.091 | 0.068 | -0.117 | -0.029 | 0.027 | 0.038 | -0.103 | -0.022 | -0.012 | -0.052 | 0.877* |
| | df | 151 | 147 | 143 | 145 | 143 | 141 | 143 | 146 | 148 | 92 | 143 | 140 | 108 | 151 |
| | p | 0.146 | 0.797 | 0.303 | 0.272 | 0.418 | 0.164 | 0.731 | 0.741 | 0.645 | 0.322 | 0.792 | 0.889 | 0.591 | < .001 |
| | N | 153 | 149 | 145 | 147 | 145 | 143 | 145 | 148 | 150 | 94 | 145 | 142 | 110 | 153 |
| total IgE | rho Spearmana | -0.195 | -0.106 | 0.172 | 0.317** | 0.371*** | 0.345** | 0.301** | 0.228* | 0.383*** | 0.517*** | 0.267* | 0.408*** | 0.293* | 0.124 |
| | df | 78 | 77 | 75 | 76 | 74 | 74 | 74 | 76 | 77 | 49 | 75 | 75 | 65 | 78 |
| | p | 0.083 | 0.351 | 0.135 | 0.005 | < .001 | 0.002 | 0.008 | 0.045 | < .001 | < .001 | 0.019 | < .001 | 0.016 | 0.273 |
| | N | 80 | 79 | 77 | 78 | 76 | 76 | 76 | 78 | 79 | 51 | 77 | 77 | 67 | 80 |
| BAT negative | rho Spearmana | 0.016 | 0.093 | -0.023 | 0.112 | 0.021 | -0.060 | -0.006 | -0.046 | -0.088 | -0.046 | 0.045 | -0.135 | 0.004 | 0.085 |
| | df | 137 | 134 | 133 | 134 | 134 | 132 | 133 | 133 | 135 | 93 | 131 | 127 | 101 | 136 |
| | p | 0.848 | 0.281 | 0.795 | 0.195 | 0.809 | 0.491 | 0.945 | 0.594 | 0.304 | 0.658 | 0.607 | 0.126 | 0.967 | 0.321 |
| | N | 139 | 136 | 135 | 136 | 136 | 134 | 135 | 135 | 137 | 95 | 133 | 129 | 103 | 138 |
| BAT positive | rho Spearmana | -0.151 | -0.014 | -0.040 | -0.036 | -0.069 | 0.070 | -0.093 | 0.014 | -0.008 | 0.011 | -0.006 | 0.016 | -0.040 | -0.085 |
| | df | 148 | 144 | 142 | 144 | 142 | 140 | 142 | 144 | 146 | 93 | 141 | 137 | 107 | 147 |
| | p | 0.066 | 0.862 | 0.636 | 0.668 | 0.409 | 0.410 | 0.268 | 0.864 | 0.927 | 0.918 | 0.948 | 0.853 | 0.678 | 0.303 |
| | N | 150 | 146 | 144 | 146 | 144 | 142 | 144 | 146 | 148 | 95 | 143 | 139 | 109 | 149 |
| BAT wasp | rho Spearmana | -0.062 | -0.019 | -0.365*** | -0.162* | -0.212* | -0.038 | -0.337*** | 0.154 | 0.456*** | 0.342*** | -0.280*** | 0.424*** | 0.215* | -0.097 |
| | df | 152 | 148 | 144 | 146 | 144 | 142 | 144 | 147 | 149 | 93 | 144 | 140 | 108 | 151 |
| | p | 0.447 | 0.814 | < .001 | 0.049 | 0.010 | 0.650 | < .001 | 0.060 | < .001 | < .001 | < .001 | < .001 | 0.024 | 0.232 |
| | N | 154 | 150 | 146 | 148 | 146 | 144 | 146 | 149 | 151 | 95 | 146 | 142 | 110 | 153 |
| BAT honeybee | rho Spearmana | -0.022 | 0.062 | 0.495*** | 0.339*** | 0.322*** | 0.165* | 0.299*** | 0.054 | -0.442*** | -0.272** | 0.448*** | -0.357*** | -0.028 | 0.038 |
| | df | 152 | 148 | 144 | 146 | 144 | 142 | 144 | 147 | 149 | 93 | 144 | 140 | 108 | 151 |
| | p | 0.783 | 0.448 | < .001 | < .001 | < .001 | 0.047 | < .001 | 0.514 | < .001 | 0.008 | < .001 | < .001 | 0.768 | 0.638 |
| | N | 154 | 150 | 146 | 148 | 146 | 144 | 146 | 149 | 151 | 95 | 146 | 142 | 110 | 153 |
| BASO BAT | rho Spearmana | 0.042 | -0.081 | 0.025 | -0.001 | 0.095 | 0.173* | 0.044 | 0.166* | 0.212** | 0.141 | -0.011 | 0.298*** | 0.233* | -0.188* |
| | df | 149 | 145 | 142 | 144 | 142 | 140 | 142 | 145 | 147 | 93 | 141 | 138 | 107 | 148 |
| | p | 0.607 | 0.331 | 0.770 | 0.992 | 0.255 | 0.040 | 0.600 | 0.045 | 0.009 | 0.172 | 0.899 | < .001 | 0.015 | 0.021 |
| | N | 151 | 147 | 144 | 146 | 144 | 142 | 144 | 147 | 149 | 95 | 143 | 140 | 109 | 150 |

***Attention.*** *** p < .05, ** p < .01, *** p < .001**

| | | TRYP | NEUT | EOS | BASO | WBC | BAT negative | BAT positive | BAT wasp | BAT honeybee | BASO W BAT |
|---|---|---|---|---|---|---|---|---|---|---|---|
| TRYP | rho Spearmana | — | | | | | | | | | |
| | df | — | | | | | | | | | |
| | p | — | | | | | | | | | |
| | N | — | | | | | | | | | |
| NEUT | rho Spearmana | 0.033 | — | | | | | | | | |
| | df | 147 | — | | | | | | | | |
| | p | 0.691 | — | | | | | | | | |
| | N | 149 | — | | | | | | | | |
| EOS | rho Spearmana | 0.030 | -0.030 | — | | | | | | | |
| | df | 147 | 151 | — | | | | | | | |
| | p | 0.713 | 0.712 | — | | | | | | | |
| | N | 149 | 153 | — | | | | | | | |
| BASO | rho Spearmana | -0.076 | 0.134 | 0.165* | — | | | | | | |
| | df | 147 | 151 | 151 | — | | | | | | |
| | p | 0.358 | 0.100 | 0.041 | — | | | | | | |
| | N | 149 | 153 | 153 | — | | | | | | |
| WBC | rho Spearmana | -0.021 | 0.877*** | 0.145 | 0.200* | — | | | | | |
| | df | 147 | 151 | 151 | 151 | — | | | | | |
| | p | 0.797 | < .001 | 0.074 | 0.013 | — | | | | | |
| | N | 149 | 153 | 153 | 153 | — | | | | | |
| BAT negative | rho Spearmana | 0.093 | 0.085 | -0.029 | -0.097 | 0.069 | — | | | | |
| | df | 134 | 136 | 136 | 136 | 136 | — | | | | |
| | p | 0.281 | 0.321 | 0.734 | 0.260 | 0.422 | — | | | | |
| | N | 136 | 138 | 138 | 138 | 138 | — | | | | |
| BAT positive | rho Spearmana | -0.014 | -0.085 | 0.181* | 0.007 | -0.064 | -0.016 | — | | | |
| | df | 144 | 147 | 147 | 147 | 147 | 137 | — | | | |
| | p | 0.862 | 0.303 | 0.027 | 0.932 | 0.437 | 0.848 | — | | | |
| | N | 146 | 149 | 149 | 149 | 149 | 139 | — | | | |
| BAT wasp | rho Spearmana | -0.019 | -0.097 | 0.108 | 0.013 | -0.083 | -0.007 | 0.504*** | — | | |
| | df | 148 | 151 | 151 | 151 | 151 | 137 | 148 | — | | |
| | p | 0.814 | 0.232 | 0.184 | 0.874 | 0.307 | 0.938 | < .001 | — | | |
| | N | 150 | 153 | 153 | 153 | 153 | 139 | 150 | — | | |
| BAT honeybee | rho Spearmana | 0.062 | 0.038 | 0.081 | -0.205* | -0.021 | 0.477*** | -0.008 | -0.270*** | — | |
| | df | 148 | 151 | 151 | 151 | 151 | 137 | 148 | 152 | — | |
| | p | 0.448 | 0.638 | 0.318 | 0.011 | 0.793 | < .001 | 0.921 | < .001 | — | |
| | N | 150 | 153 | 153 | 153 | 153 | 139 | 150 | 154 | — | |
| BASO BAT | rho Spearmana | -0.081 | -0.188* | 0.121 | 0.437*** | -0.162* | -0.124 | -0.064 | 0.067 | -0.101 | — |
| | df | 145 | 148 | 148 | 148 | 148 | 136 | 146 | 149 | 149 | — |
| | p | 0.331 | 0.021 | 0.140 | < .001 | 0.048 | 0.147 | 0.437 | 0.412 | 0.217 | — |
| | N | 147 | 150 | 150 | 150 | 150 | 138 | 148 | 151 | 151 | — |
