# Supplementary material for: Association between results of component-resolved diagnostics and basophil activation in Hymenoptera venom allergy: A registry-based cross-sectional study in adults
Source: PLoS One. 2026 Jun 17;21(6):e0350189. doi: 10.1371/journal.pone.0350189 (PMC13274868; doi:10.1371/journal.pone.0350189)
Supplement: S3 Table — Distribution of missing data in the study dataset. (PDF) [file pone.0350189.s003.pdf]

S3 Table. Distribution of missing data in the study dataset.

Variable	Group A n = 27		Group B n = 32		Group C n = 95	
	n	[%]	n	[%]	n	[%]
**Tryptase**	3	11.1	0	0	1	1.1
**sIgE honeybee**	2	7.4	2	6.2	4	4.2
**sIgE wasp**	2	7.4	4	12.5	6	6.3
**rApi m 1**	1	3.7	1	3.1	6	6.3
**rApi m 2**	0	0	1	3.1	5	5.3
**rApi m 3**	2	7.4	2	6.2	4	4.2
**rApi m 5**	2	7.4	2	6.2	6	6.3
**rApi m 10**	1	3.7	2	6.2	5	5.3
**rVes v 1**	0	0	2	6.2	3	3.2
**rVes v 5**	0	0	1	3.1	2	2.1
**BAT positive control**	0	0	1	3.1	3	3.2
**BAT negative control**	6	22.2	5	16	4	4.2
