# Supplementary material for: Association between results of component-resolved diagnostics and basophil activation in Hymenoptera venom allergy: A registry-based cross-sectional study in adults
Source: PLoS One. 2026 Jun 17;21(6):e0350189. doi: 10.1371/journal.pone.0350189 (PMC13274868; doi:10.1371/journal.pone.0350189)
Supplement: Attachment — Submitted filename: Response to Reviewers.pdf [file pone.0350189.s004.pdf]

**Warsaw, March 9th, 2025**

**Dear Editor and Reviewers,**

On behalf of my co-authors, I am glad to submit our revised manuscript entitled *"Association between results of component-resolved diagnostics and basophil activation in Hymenoptera venom allergy: a registry-based cross-sectional study in adults"* for publication in **PLOS ONE**. We thank the Reviewers for the careful assessment of our manuscript and for their precise feedback.

We would also like to provide additional information and responses to the following issues which have been raised by the Reviewers.

*2. Thank you for stating the following in the Acknowledgments Section of your manuscript:*

*"The authors received no specific funding for the presented work."*

*We note that you have provided funding information that is not currently declared in your Funding Statement. However, funding information should not appear in the Acknowledgments section or other areas of your manuscript. We will only publish funding information present in the Funding Statement section of the online submission form.*

*Please remove any funding-related text from the manuscript and let us know how you would like to update your Funding Statement. Currently, your Funding Statement reads as follows:*

*"The author(s) received no specific funding for this work."*

*Please include your amended statements within your cover letter; we will change the online submission form on your behalf.*

According to the Reviewer's suggestion, we have removed funding-related text from the manuscript.

*Reviewer #2: The author has carried out a well-designed, registry-based, cross-sectional study evaluating the association between component-resolved diagnostics (CRD) and basophil activation testing (BAT) in adults with Hymenoptera venom allergy. It is clinically relevant and addresses a clear diagnostic challenge in allergy and immunology. The use of clearly defined patient groups, and integration of CRD with functional testing are notable strengths of this study. This study addresses an important diagnostic issue in venom allergy and presents clinically meaningful findings, but the author needs to address some minor clarifications in methodology, and interpretation of results.*

*1.Lines 45–46, 317–320: Statements that CRD and BAT 'enhance accuracy' or 'enhance sensitivity and specificity' imply improved diagnostic performance. Given the retrospective, cross-sectional design of the study, I recommend rephrasing these statements to emphasize association or supportive clinical use rather than diagnostic enhancement.*

We agree and have rephrased lines 45-46 and 317-320 accordingly.

*2.Lines 68–72, 79–82: The discussion of limitations of conventional diagnostics and the potential benefits of CRD and BAT overlaps conceptually and leaves some speculations regarding improved subcutaneous immunotherapy qualification. I suggest condensing these sections and clarifying that the statements reflect hypotheses or emerging evidence rather than established clinical practice.*

According to the suggestion, we have condensed lines 68-72 and 79-82 in the revised version in order to improve clarity.

*3.Lines 104–107: Patients in groups B and C were subdivided by reaction severity without a clear explanation of the rationale. I recommend to briefly clarify how this stratification aligns with the study objectives.*

In the revised version, we have provided a brief explanation of the rationale. This was intended to enable the assessment of associations between BAT and CRD results and the severity of allergic symptoms.

*4.Lines 120–127, 321–326: BAT was conducted using a single venom concentration without dose–response testing. I recommend acknowledging this limitation explicitly in the limitations section and discussing its potential impact on interpretation of basophil sensitivity.*

We agree and have provided a short explanation in the limitations section of the revised manuscript.

*5.Lines 150–151: No correction for multiple testing was applied despite numerous correlation analyses. I recommend providing a clearer justification for this decision, even within an exploratory framework.*

As the correlation analyses were exploratory in nature and aimed at identifying potential associations between laboratory parameters, we chose not to apply a strict correction, which could substantially increase the risk of type II error in this relatively moderate sample size. The results should therefore be interpreted as hypothesis-generating rather than confirmatory. This clarification has been added to the Methods section.

*6.Lines 169–172: Missing data are reported, but their potential impact on bias or statistical power is not discussed. I suggest briefly stating whether the missing data was assumed to be at random.*

In our opinion, the proportion of missing data was low and distributed across the study groups without a discernible pattern. The missing values were primarily due to technical or logistical reasons (e.g., insufficient sample material), and were therefore assumed to be missing at random. This clarification has been added to the Discussion section.

*Reviewer #3: This registry-based cross-sectional study analyzes the utility of the Basophil Activation Test (BAT) and Component-Resolved Diagnostics (CRD) in 154 patients*

*evaluated for Hymenoptera venom allergy. The topic is clinically relevant, particularly regarding the specific sensitization profiles in Central Europe. (In addition, when considering the therapeutic potential of venom extracts, it can also give an insight in clinical context.) The manuscript presents interesting data regarding the correlation between specific IgE (sIgE) components and BAT reactivity. However, the manuscript requires revision before it can be accepted. There are gaps in the reporting of methodology (specifically the BAT protocol and patient grouping criteria), discrepancies in the results (missing data in tables, conflicting statistical significance), and issues with data availability statements. Furthermore, the figures and tables require reorganization to improve readability and standard scientific formatting. Below are my specific comments arranged by section.*

*#Major Revisions 1. There is a contradiction regarding data access. The metadata states "No - some restrictions will apply", yet the text under Data Availability states "All relevant data have been included within the paper and its Supporting Information files". If the data is fully available in S1 Table, the metadata should be updated to "Yes." If restrictions apply, the text must explain them.*

We have revised the metadata in order to avoid this contradiction.

*2. The Methods section states that participants were divided into three groups (A, B, C) "according to the final diagnosis and qualification for treatment". However, the specific clinical criteria used to make these assignments are missing. - The definition for Group A is currently located in the Introduction ("absence of confirmed venom allergy") rather than the Methods. Please provide the definition or criteria of group A, B and C to the Methods. Please define what constitutes "absence of confirmed allergy" (e.g., negative skin tests? negative sIgE?) and state the criteria (e.g., skin test reaction size, sIgE thresholds) used to categorize a patient into the Bee group vs. the Wasp group, also shown in Table 6 which separates patients based on qualification for Bee vs. Wasp immunotherapy.*

Group A consisted of patients who were not diagnosed with venom allergy, defined as the absence of venom-specific IgE antibodies and a negative basophil activation test. The threshold for sIgE was 0.35 IU/mL. Group B consisted of patients who were qualified for bee venom immunotherapy, and Group C consisted of patients who were qualified for wasp venom immunotherapy, respectively. The aforementioned information has been incorporated into the revised version of the manuscript.

*3. Missing Methodological Details (BAT Protocol)** The "Laboratory procedures" section can be strengthened by adding the detail required for replication. - In line 122 and line 128, the duration of the incubation is not stated. - In line 128, it is unclear if staining occurred simultaneously with stimulation or as a subsequent step. Please specify the time and temperature conditions for the antibody staining. - Was a stopping solution (e.g., EDTA) used to halt the reaction?*

We have improved the “Laboratory procedures” section profoundly according to the advice. The incubation consisted of two steps: 25 minutes in an air incubator, followed by 20 minutes at 2–8 °C. Staining was performed as a subsequent step after stimulation. Twenty µL of staining reagent was added to each tube. Samples were then incubated for 20 minutes at 2–8 °C. No stopping solution was used. Immediately after the staining step, a lysing solution was added. After 5 minutes—once red blood cells were lysed—the samples were centrifuged, the

supernatant was removed, and the pellet was resuspended in 200 µL of PBS. The samples were then analyzed immediately using a flow cytometer.

*4. Please add the row for rVes v 5 to Table 3, which is omitted despite the text identifying it as a major component with significant correlations.*

We have added the missing row in the revised version.

*5.Figure 3 identifies a patient with double-positive reactivity. Please clarify in the Methods or Results section which group (B or C) this patient was assigned to, or if they were treated as a separate entity, as this affects the specificity analysis of the respective groups.*

This result is presented mainly as a didactic example of an unusual phenomenon. The patient referred to in this comment was classified into Group B, as wasp venom allergy was considered the primary cause of anaphylaxis in this case and constituted the target of immunotherapy. We have provided additional clarification in the Results section.

*6. The Results section states that "nonspecific reactivity assessed by the positive control did not differ significantly" among groups. However, Table 3 reports a p-value of 0.046 for "BAT control pos.", which is statistically significant (<0.05). Please clarify this discrepancy.*

While the overall ANOVA/Kruskal–Wallis test showed a p-value of 0.046 for the BAT positive control, none of the post hoc pairwise comparisons reached statistical significance. Therefore, although the global test suggested a borderline difference, no significant differences were identified between specific groups. To avoid confusion, we have revised the Results section to reflect this more precisely.

*7. The Conclusion section need to be improved by addressing the study objectives mentioned in the Introduction section. - The conclusion focuses on specific molecules (Api m 1, Ves v 5) but does not explicitly relate the findings back to the differentiation of the three study groups (A, B, C) as proposed in the primary objective. - In line 87-88, "The secondary objective was to analyze the association between the investigated parameters and the severity of anaphylactic reactions in the medical history of participants." I suggest to state even the negative finding, no association, in the Conclusion.*

The Conclusion has been reformulated to better align with the objectives stated in the Introduction, including both the group differentiation analysis and the negative findings regarding reaction severity.

*#Minor Revisions 1.In Methods Section, - In "Setting and study participants", please replace "included all adult patients" with the specific number (n=154) to provide immediate context on the study power. - There is potential confusion regarding consent. It stated "individuals unable to provide informed consent were not referred", but later says consent was "waived". Please clarify that patients lacking legal capacity for _clinical_ consent were excluded, which is distinct from the _research_ consent waiver. - Please mention the*

*approximate volume of blood collected to indicate the sample requirement for this battery of tests.*

We have implemented changes according to the suggestions. Approximately 10–15 mL of peripheral venous blood was collected from each participant to perform all laboratory analyses.

*2.In Result Section - I suggest clarifying the sentence ""BAT results were significantly higher for the venom of culprit insect" for general readers.*

We have changed “BAT results” to “basophil reactivity” in order to clarify the sentence.

*3. In Discussion Section - In Discussion section, please remove the bulleted list of limitations and rewrite this as a continuous narrative paragraph. - In the Discussion, the statement "few previous reports have examined..." requires citations (e.g., referencing Eberlein et al. or Korošec et al.).*

The Discussion has been rewritten in accordance with the Reviewer’s advice. We have added suggested citations.

*4.In Figures - I suggest to merge figure 1 and 2. These figures show the same groups responding to different stimuli. It would be much clearer to merge them into a single figure with two panels ((A): Wasp stimulation, (B) Honeybee stimulation). Ensure Y-axes have identical scales and titles. - In figure 3, please add row labels (A, B, C, D) directly to the image and update the caption to match. The current description ("Following lines from top to bottom") is difficult to follow. - In figure 4, please add clear axis titles (e.g., "sIgE Components", "Basophil Activation") to make the heatmap self-explanatory.*

Figures 1 and 2 have been merged into a single figure with two panels. The row labels have been added to Figure 3, and the captions have been updated. We have also provided additional axis titles.

*5.In tables. - The URL citation in the title of Table 1 should be converted to a formal reference. I also suggest to consider to move this table to a more proper location not to disrupts the Introduction or I'd like to additional explanation why only eight allergen picked in this study. - I suggest to reorganize table 2 and 3 into one with patient demographics(combining age and sex) and the other with laboratory findings(Tryptase, cells, slgE, BAT). - 'Table 4. Distribution of missing data' disrupts the flow of the results. I suggest to merge it to the new table with laboratory findings or move it to Supplementary Information. - The readability of Table 5 can be improved. Please regroup the columns so that **rho** and **p-value** are listed side-by-side under their respective headers (BAT Wasp / BAT Honeybee). - All abbreviations used in tables (e.g., SD, Med, NEUT) must be defined in the table footnotes.*

We have converted the URL citation to a formal reference. We have also moved the aforementioned table to the Methods section. Additional explanation regarding the chosen allergens has been provided in the revised version of the manuscript. According to the suggestion, we have merged Table 2 and Table 3 into one, and we have also transferred Table

4 to the Supporting Information. The columns of Table 5 have been regrouped for clarity. Missing abbreviations have been defined in the table footnotes.

*6. Style - Please unify the use of abbreviation throughout the manuscript. The full name (e.g. Apis mellifera for Api m) should be defined upon first use in the body text and the abbreviation should be use consistently after it is defined (e.g. sIgE)*

We have unified abbreviations throughout the revised manuscript and have added missing full names.

*Reviewer #4: The manuscript entitled "Association between results of component-resolved diagnostics and basophil activation in Hymenoptera venom allergy" reports on a retrospective cross-sectional study that evaluates the association between component-resolved diagnostics (CRD) and the basophil activation test (BAT) in 154 adults with Hymenoptera venom allergy. Patients were divided into three groups: not qualified for subcutaneous immunotherapy (SCIT), qualified for bee venom SCIT, and qualified for wasp venom SCIT. The key findings include moderate correlations between BAT results and sIgE levels to Api m 1 and Ves v 5, no association between test parameters (BAT, sIgE) and the severity of prior sting reactions (Müller grades), and that CRD and BAT may provide complementary diagnostic information, particularly in cases of polysensitization or ambiguous conventional test results. The study is well-designed, with appropriate statistical methods and a balanced discussion of limitations. However, minor revisions are needed to clarify the study rationale and clinical implications, ensuring accessibility for PLOS ONE's broad readership. Minor Revisions Required 1.Abstract Issue: The problem statement is underdeveloped. The rationale for comparing CRD and BAT is not explicitly stated. Please explain the interest to compare these two methods in the diagnostic and therapeutic management of HVA patients.*

We have rephrased the Background of the Abstract in order to develop the problem statement more specifically.

*Refine the clinical implications: I do not think one can assert "enhances diagnostic accuracy" in the conclusion since the study objectives were not focused on diagnostic accuracy (sensitivity, specificity etc.). Instead, I would suggest replacing it with something like: CRD and BAT provide complementary information, particularly in cases of polysensitization or ambiguous conventional test results, potentially improving patient selection for SCIT.*

We have added these suggestions to the revised Abstract, namely to the Background and the Conclusions.

*2.Introduction Issue: Briefly explain SCIT indications before this sentence: "The combined use of BAT and CRD may potentially improve the accuracy of qualification for subcutaneous allergen immunotherapy (SCIT), etc." The problem statement is underdeveloped. The rationale for comparing CRD and BAT is not explicitly stated. Please explain the interest to compare these two methods in the diagnostic and therapeutic management of HVA patients.*

A brief explanation of SCIT indications has been added to the paragraph, along with a more explicit statement of the rationale for the comparison.

We respectfully suggest that our manuscript fits well within the scope of **PLOS ONE**, which values scientifically rigorous, methodologically sound studies that advance knowledge across disciplines and are relevant for clinical and translational practice.

Thank you for considering our work. We are looking forward to your feedback.

**Sincerely,**

Krzysztof Piwowarek, MD, PhD
Department of Internal Medicine, Infectious Diseases and Allergology
Military Institute of Medicine – National Research Institute
Warsaw, Poland
