# Supplementary material for: Association between results of component-resolved diagnostics and basophil activation in Hymenoptera venom allergy: A registry-based cross-sectional study in adults
Source: PLoS One. 2026 Jun 17;21(6):e0350189. doi: 10.1371/journal.pone.0350189 (PMC13274868; doi:10.1371/journal.pone.0350189)
Supplement: Attachment — Submitted filename: Response to Reviewers.docx [file pone.0350189.s005.docx]

**Warsaw, March 27th, 2026**

**Dear Editor and Reviewers,**

On behalf of all co-authors, I am glad to resubmit our revised manuscript entitled *“Association between results of component-resolved diagnostics and basophil activation in Hymenoptera venom allergy: a registry-based cross-sectional study in adults”* for publication in **PLOS ONE**. We thank the Reviewer for the meticulous assessment of our manuscript and for the precise feedback, which motivated us to improve our work.

We would also like to provide additional information and necessary responses to the following issues which have been raised by the Reviewer.

*In my view, the manuscript still attributes more certainty to the results than the design permits. A retrospective cross-sectional study can show that certain markers are associated with BAT results in this sample, but it cannot establish that one marker exerts the strongest impact on diagnosis or that it has a decisive biological role. Expressions such as influence, impact, and decisive role suggest causal hierarchy or clinical superiority. The data presented support a more limited statement, namely that Api m 1 and Ves v 5 were the markers most strongly correlated with BAT among the variables examined.*

We agree that the retrospective cross-sectional design of our study does not allow for establishing hierarchical or decisive roles of individual markers. In response, we have carefully revised the manuscript throughout to avoid language suggesting causality or clinical superiority. Specifically, terms such as “impact,” “influence,” and “decisive role” have been removed or replaced with more appropriate expressions such as “correlation”. These changes have been implemented across the Abstract, Results, and Discussion sections to ensure consistency with the study design and to avoid overinterpretation.

*The way the groups are defined leaves unresolved circularity. Group A is characterized in part by negative venom-specific IgE and negative BAT, yet the manuscript does not specify in operational terms how patients were assigned to the bee and wasp immunotherapy groups. If clinical history, skin testing, extract-specific IgE, BAT, or component-resolved diagnostics contributed to assignment, and those same variables are then compared across groups as if they were independent outcomes, the observed differences may largely reproduce the initial classification logic. The manuscript should therefore provide a stepwise diagnostic algorithm showing which criteria determined group membership and which measures were reserved for comparative analysis.*

We thank the Editor for this important methodological comment. We agree that the original version of the manuscript did not sufficiently clarify how patients were assigned to study groups and how this relates to the variables analyzed, which could raise concerns about circularity.

In response, we have revised the Methods section of manuscript to include a detailed stepwise diagnostic algorithm describing:

- the role of clinical history and serum venom extract-specific IgE in the initial assessment of sensitization
- the criteria used for qualification to bee or wasp venom immunotherapy
- the role of component-resolved diagnostics (CRD) and the basophil activation test (BAT) as complementary investigations

In addition, we have addressed this issue in the Limitations section, where we explicitly acknowledge that some of the diagnostic measures analyzed, particularly CRD, contributed to routine clinical decision-making. Therefore, between-group comparisons are presented as descriptive, and some degree of circularity cannot be excluded. These revisions were made to ensure transparent reporting of the diagnostic workflow and to avoid overinterpretation of between-group differences.

*The ethics wording remains difficult to reconcile. The text states that individuals unable to provide informed consent were excluded, but also states that written informed consent was waived because the study was retrospective and anonymized. As written, it is not clear whether the exclusion referred to inability to consent to routine clinical procedures, inability to consent to research participation, or something else. There is also a chronology issue that should be addressed explicitly, since the patient evaluation period ended before the stated ethics approval date. If the approval covered retrospective analysis of already collected data, the manuscript should say that directly and unambiguously.*

In order to avoid unambiguity, we have revised both the Ethics statement and the Methods section to ensure consistency and transparency. We have clarified that the study represents a retrospective analysis of previously collected clinical data, and that the Bioethics Committee approval covered this retrospective use of anonymized data. We have revised the exclusion criteria to clearly indicate that they refer to the inability to provide informed consent to routine clinical diagnostic procedures or venom immunotherapy, rather than to research participation. We have highlighted difference between the patient evaluation period and the later data extraction and analysis phase, which took place after ethics approval.

*The statistical approach is not sufficiently guarded in relation to the number of analyses performed. The manuscript labels the work exploratory, but it still interprets multiple nominal p values below 0.05 as positive findings across a broad set of tests and correlations. In that setting, isolated borderline results can arise from multiplicity alone. Several of the reported associations fall very close to the conventional threshold, which limits confidence in their stability. A stricter presentation would either apply a multiple-testing correction or state clearly that these p values are descriptive and hypothesis-generating rather than confirmatory.*

We agree with the Editor that, given the number of analyses performed, the results should be interpreted with appropriate caution. To address this, we have revised the manuscript to explicitly state that:

- the analyses are exploratory in nature
- the reported p-values are descriptive and hypothesis-generating rather than confirmatory
- findings, particularly those with p-values close to the conventional threshold, should be interpreted cautiously in the context of multiple comparisons

We have not applied formal multiple-testing correction, as the primary aim of the study was exploratory; however, this limitation is now clearly acknowledged and reflected in the Methods section.

*Some reported summary statistics appear inconsistent enough to require a full audit of the tables. The neutrophil data are one example, where the means are presented in the thousands but the standard deviations are around 1.26 and 1.27, which suggests a unit mismatch, formatting error, or incorrect transcription. Another problematic entry is the severe bee subgroup value for rApi m 3, where the reported mean, standard deviation, and median do not appear to form a plausible combination. These inconsistencies should be checked against the raw dataset and the statistical output, with corrections made wherever necessary.*

We thank the Editor for carefully identifying these inconsistencies. We have performed an additional audit of the dataset, statistical outputs, and reported summary statistics.

This review revealed two issues:

1. **Neutrophil counts:**
   In some table cells, neutrophil values were inadvertently reported using different unit conventions (absolute counts vs. values expressed in thousands), which resulted in impossible combinations of means and standard deviations. All neutrophil data have now been standardized to a single, consistent unit.
2. **rApi m 3 values (severe anaphylaxis honeybee subgroup):**
   The previously reported standard deviation and median were mistakenly transposed. This error has now been corrected based on the original statistical output.

We regret these errors and appreciate the opportunity to correct them. These changes do not materially affect the overall results or conclusions of the study.

*The mechanistic explanation offered for Api m 1 and Ves v 5 is not well grounded in the underlying biology. The manuscript suggests that their relatively low molecular weight may increase accessibility to FcεRI receptors, but FcεRI binds IgE already attached to the basophil surface, not the allergen itself. Basophil activation depends on allergen-mediated cross-linking of receptor-bound IgE, and that process is shaped by factors such as sensitization profile, epitope architecture, and functional binding characteristics. The proposed explanation is therefore speculative and should not be presented as if it were a supported interpretation of the current results.*

We agree with the concerns raised regarding the mechanistic interpretation. In response, we have **removed the speculative explanation** suggesting that the relatively low molecular weight of Api m 1 and Ves v 5 may increase accessibility to FcεRI receptors. We agree that this interpretation was not adequately supported by current knowledge of basophil activation mechanisms. The revised manuscript no longer includes this statement.

*The severity analysis should be stated more carefully. The absence of a statistically significant association in these subgroups does not justify a firm conclusion that severity is unrelated to the measured markers. The bee subgroup is especially limited because the mild-reaction category contains very few patients compared with the severe category, and that imbalance reduces the ability to detect anything other than large differences. The manuscript should therefore report that no association with severity was detected in this dataset, rather than presenting the result as evidence against any relationship.*

We agree that the interpretation of the severity analysis should be more cautious. In response, we have revised the manuscript to avoid any statements suggesting the firm absence of a relationship between the investigated markers and reaction severity. The Discussion and Conclusions sections have been updated to state that **no statistically significant association was detected in this dataset,** rather than implying a lack of association. We have also emphasized the limited size of certain subgroups, particularly the honeybee subgroup with a small number of patients in the mild-reaction category, which reduces the ability to detect anything other than large differences.

We respectfully suggest that our manuscript fits well within the scope of **PLOS ONE**, which values scientifically rigorous, methodologically sound studies that advance knowledge across disciplines and are relevant for clinical and translational practice.

Thank you for considering our work. We are looking forward to your feedback.

**Sincerely,**

Krzysztof Piwowarek, MD, PhD
Department of Internal Medicine, Infectious Diseases and Allergology
Military Institute of Medicine – National Research Institute
Warsaw, Poland
