# Supplementary material for: Association between results of component-resolved diagnostics and basophil activation in Hymenoptera venom allergy: A registry-based cross-sectional study in adults
Source: PLoS One. 2026 Jun 17;21(6):e0350189. doi: 10.1371/journal.pone.0350189 (PMC13274868; doi:10.1371/journal.pone.0350189)
Supplement: Attachment — Submitted filename: Response_to_Reviewers_auresp_3.docx [file pone.0350189.s006.docx]

**Warsaw, May 7th, 2026**

**Dear Editor and Reviewers,**

On behalf of all co-authors, I am glad to resubmit our revised manuscript entitled *“Association between results of component-resolved diagnostics and basophil activation in Hymenoptera venom allergy: a registry-based cross-sectional study in adults”* for publication in **PLOS ONE**. We thank the Reviewer for the profound assessment of our manuscript and for the feedback, which has improved our work.

We would also like to provide necessary information and responses to the following issues which have been raised by the Reviewer.

*“The manuscript was improved and the revisions were made in a correct direction, but several of them were incomplete. In my view, the main remaining concerns are the ones that still limit interpretation even after revision, together with practical fixes that could be implemented without additional wet lab work.*

*The severity analysis remains a key weakness. The authors now acknowledge that some subgroups were small, especially the honeybee mild-reaction subgroup, and that the findings therefore do not justify a firm conclusion. That is an improvement, but the analysis is still underpowered for a confident statement about severity-related associations. I would therefore encourage the authors to tone this down even further in the abstract, discussion, and conclusions, and to present the severity results explicitly as inconclusive subgroup analyses rather than negative findings. It would also strengthen the manuscript if the authors reported effect sizes and confidence intervals, not only p values, so that readers can better judge the uncertainty around these estimates.”*

We agree that the severity analysis is underpowered, particularly in the honeybee mild-reaction subgroup, and does not allow for firm conclusions. In response, we have further revised the Abstract, Discussion, and Conclusions to explicitly present these results as inconclusive subgroup analyses. Additionally, we have now reported 95% confidence intervals for all severity-related analyses to better reflect the magnitude and uncertainty of the estimates. We believe these changes improve the transparency and interpretability of the results.

*“Circularity remains a substantive methodological limitation. The authors added a clearer diagnostic algorithm and now acknowledge that some circularity cannot be excluded because component-resolved diagnostics contributed to routine clinical decision-making. This improves transparency, but it does not remove the issue. I would encourage the authors to distinguish more clearly which variables were used for clinical classification and which were treated as analytic outcomes, and then to frame the between-group comparisons as descriptive only. If possible, the authors could also add a sensitivity analysis excluding variables most directly involved in treatment assignment, or at least provide a table that identifies classification-related versus evaluation-only variables.”*

To improve transparency and address potential circularity, we have now clearly distinguished variables used for clinical classification from those treated as analytic outcomes. This distinction has been summarized in a new table (Table 5), where variables are categorized as classification-related or evaluation-only.

*“The statistical analysis part is still not very strong. The revision presents the findings more cautiously, but the manuscript still relies on multiple comparisons and nominal significance testing. A practical improvement, without requiring new experiments, would be to restructure the Results around effect sizes, confidence intervals, and a smaller set of prespecified primary comparisons, while moving more exploratory correlations to the Supplement. Alternatively, if the authors prefer to retain the present structure, the manuscript should state even more explicitly that these analyses are hypothesis-generating rather than confirmatory.”*

We agree and in addition to revising the statistical analysis section, we have now explicitly emphasized in the Discussion that the aforementioned analyses are exploratory and hypothesis-generating rather than confirmatory.

*“The retrospective single-center design remains an important limitation for generalizability. The authors now acknowledge this more clearly, which is appropriate, but the claims should remain tightly aligned with what this dataset can actually support. I would therefore suggest that the authors narrow the wording where needed and avoid implying broader diagnostic applicability beyond similar referral populations.”*

We agree that the retrospective single-center design limits the generalizability of our findings. In response, we have further revised the manuscript to ensure that the conclusions remain closely aligned with the scope of the dataset and do not imply broader diagnostic applicability beyond similar referral populations.

*“The absence of skin test data and the use of only a single BAT concentration still weaken the diagnostic interpretation. Since this cannot be remedied experimentally at this stage, I would encourage the authors to address it more explicitly in the manuscript by clarifying how these limitations constrain interpretation, and by avoiding any implication that BAT performance or thresholds were comprehensively characterized here. A brief supplementary flowchart or summary table showing which diagnostic modalities were available for how many patients would also improve transparency.”*

We agree that the absence of skin test data and the use of a single BAT concentration limit the diagnostic interpretation of our findings. In response, we have further clarified these limitations in the Discussion, explicitly stating how they constrain interpretation and avoiding any implication that BAT performance or diagnostic thresholds were comprehensively characterized. We would also like to notice that we have already included a supplementary file summarizing data completeness, which details the availability of diagnostic modalities across the study population.

*“Missing data remain a lesser but still relevant concern. The explanation provided by the authors is reasonable, but the manuscript would be stronger if the authors added a compact supplementary table showing missingness by variable and group, together with a brief statement on whether complete-case analyses differed materially from the full analytic sample.”*

We have already included a supplementary table (S3 Table) summarizing missing data by variable and group, which provides an overview of data availability across the study population. In addition, we have included a brief statement in the revised manuscript addressing whether complete-case analyses differed materially from the full analytic sample.

*“Some conclusions also still extend slightly beyond what the design can firmly support. I would encourage the authors to make the final take-home message as precise as possible, emphasizing association and complementary diagnostic information rather than clinical utility in a stronger sense. Statements about decision support would be better framed as requiring prospective validation.”*

We agree that the conclusions should remain closely aligned with the scope of the study design. In response, we have revised the Conclusions section to ensure a more precise and cautious message, emphasizing associations and complementary diagnostic information as well as excluding broader clinical utility. We believe these changes improve the accuracy and balance of the manuscript.

We respectfully suggest that our manuscript fits within the scope of **PLOS ONE**, which values methodologically sound studies that advance knowledge and are relevant for clinical and translational practice.

Thank you for considering our work. We are looking forward to your feedback.

**Sincerely,**

Krzysztof Piwowarek, MD, PhD
Department of Internal Medicine, Infectious Diseases and Allergology
Military Institute of Medicine – National Research Institute
Warsaw, Poland
