# Supplementary material for: Association between results of component-resolved diagnostics and basophil activation in Hymenoptera venom allergy: A registry-based cross-sectional study in adults
Source: PLoS One. 2026 Jun 17;21(6):e0350189. doi: 10.1371/journal.pone.0350189 (PMC13274868; doi:10.1371/journal.pone.0350189)
Supplement: Peer review history

# Peer review: Decision Letter 0

**DOI:** 10.1371/journal.pone.0350189.r001

**Authors:** Petr Heneberg

27 Jan 2026

PONE-D-25-68655

Association between results of component-resolved diagnostics and basophil activation in Hymenoptera venom allergy: a registry-based cross-sectional study in adults.

PLOS One

Dear Dr. Piwowarek,

Thank you for submitting your manuscript to PLOS ONE. After careful consideration, we feel that it has merit but does not fully meet PLOS ONE’s publication criteria as it currently stands. Therefore, we invite you to submit a revised version of the manuscript that addresses the points raised during the review process.

Please submit your revised manuscript by Mar 13 2026 11:59PM. If you will need more time than this to complete your revisions, please reply to this message or contact the journal office at plosone@plos.org. When you're ready to submit your revision, log on to https://www.editorialmanager.com/pone/ and select the 'Submissions Needing Revision' folder to locate your manuscript file.

Please include the following items when submitting your revised manuscript:

- A letter that responds to each point raised by the academic editor and reviewer(s). You should upload this letter as a separate file labeled 'Response to Reviewers'.
- A marked-up copy of your manuscript that highlights changes made to the original version. You should upload this as a separate file labeled 'Revised Manuscript with Track Changes'.
- An unmarked version of your revised paper without tracked changes. You should upload this as a separate file labeled 'Manuscript'.

If you would like to make changes to your financial disclosure, please include your updated statement in your cover letter. Guidelines for resubmitting your figure files are available below the reviewer comments at the end of this letter.

If applicable, we recommend that you deposit your laboratory protocols in protocols.io to enhance the reproducibility of your results. Protocols.io assigns your protocol its own identifier (DOI) so that it can be cited independently in the future. For instructions see: https://journals.plos.org/plosone/s/submission-guidelines#loc-laboratory-protocols. Additionally, PLOS ONE offers an option for publishing peer-reviewed Lab Protocol articles, which describe protocols hosted on protocols.io. Read more information on sharing protocols at https://plos.org/protocols?utm_medium=editorial-email&utm_source=authorletters&utm_campaign=protocols.

We look forward to receiving your revised manuscript.

Kind regards,

Petr Heneberg

Academic Editor

PLOS One

Journal Requirements:

When submitting your revision, we need you to address these additional requirements.

1. Please ensure that your manuscript meets PLOS ONE's style requirements, including those for file naming. The PLOS ONE style templates can be found at

https://journals.plos.org/plosone/s/file?id=wjVg/PLOSOne_formatting_sample_main_body.pdf and

https://journals.plos.org/plosone/s/file?id=ba62/PLOSOne_formatting_sample_title_authors_affiliations.pdf

2. Thank you for stating the following in the Acknowledgments Section of your manuscript:

“The authors received no specific funding for the presented work.”

We note that you have provided funding information that is not currently declared in your Funding Statement. However, funding information should not appear in the Acknowledgments section or other areas of your manuscript. We will only publish funding information present in the Funding Statement section of the online submission form.

Please remove any funding-related text from the manuscript and let us know how you would like to update your Funding Statement. Currently, your Funding Statement reads as follows:

“The author(s) received no specific funding for this work.”

Please include your amended statements within your cover letter; we will change the online submission form on your behalf.

3. If the reviewer comments include a recommendation to cite specific previously published works, please review and evaluate these publications to determine whether they are relevant and should be cited. There is no requirement to cite these works unless the editor has indicated otherwise.

[Note: HTML markup is below. Please do not edit.]

Reviewers' comments:

Reviewer's Responses to Questions

**Comments to the Author**

1. Is the manuscript technically sound, and do the data support the conclusions?

The manuscript must describe a technically sound piece of scientific research with data that supports the conclusions. Experiments must have been conducted rigorously, with appropriate controls, replication, and sample sizes. The conclusions must be drawn appropriately based on the data presented.

Reviewer #1: Yes

Reviewer #2: Yes

Reviewer #3: Yes

Reviewer #4: Yes

**********

2. Has the statistical analysis been performed appropriately and rigorously?

Reviewer #1: Yes

Reviewer #2: Yes

Reviewer #3: Yes

Reviewer #4: Yes

**********

3. Have the authors made all data underlying the findings in their manuscript fully available?

The PLOS Data policy requires authors to make all data underlying the findings described in their manuscript fully available without restriction, with rare exception (please refer to the Data Availability Statement in the manuscript PDF file). The data should be provided as part of the manuscript or its supporting information, or deposited to a public repository. For example, in addition to summary statistics, the data points behind means, medians and variance measures should be available. If there are restrictions on publicly sharing data—e.g. participant privacy or use of data from a third party—those must be specified.

Reviewer #1: Yes

Reviewer #2: Yes

Reviewer #3: No

Reviewer #4: Yes

**********

4. Is the manuscript presented in an intelligible fashion and written in standard English?

PLOS ONE does not copyedit accepted manuscripts, so the language in submitted articles must be clear, correct, and unambiguous. Any typographical or grammatical errors should be corrected at revision, so please note any specific errors here.

Reviewer #1: Yes

Reviewer #2: Yes

Reviewer #3: Yes

Reviewer #4: Yes

**********

5. Review Comments to the Author

Please use the space provided to explain your answers to the questions above. You may also include additional comments for the author, including concerns about dual publication, research ethics, or publication ethics. (Please upload your review as an attachment if it exceeds 20,000 characters)

Reviewer #1: The manuscript presents a registry-based cross-sectional study on Hymenoptera venom allergy (HVA) diagnosis in 154 adults from Poland (pp. 1, 8). The authors investigated the association between component-resolved diagnostics (CRD) and the basophil activation test (BAT) to improve the identification of the culprit venom and qualification for specific immunotherapy (SCIT). Key findings indicate a moderate correlation between BAT results and sIgE to major components Api m 1 (honeybee) and Ves v 5 (wasp) (pp. 1, 9, 20). The study concludes that combining CRD and BAT enhances diagnostic accuracy, particularly in complex cases like polysensitization (pp. 9, 26). Notably, the severity of a patient's historical sting reaction did not correlate with either of the measured laboratory parameters. The manuscript is well-structured, the methodology is sound, and the results are clearly presented. I have no comments.

Reviewer #2: The author has carried out a well-designed, registry-based, cross-sectional study evaluating the association between component-resolved diagnostics (CRD) and basophil activation testing (BAT) in adults with Hymenoptera venom allergy. It is clinically relevant and addresses a clear diagnostic challenge in allergy and immunology. The use of clearly defined patient groups, and integration of CRD with functional testing are notable strengths of this study. This study addresses an important diagnostic issue in venom allergy and presents clinically meaningful findings, but the author needs to address some minor clarifications in methodology, and interpretation of results.

1.Lines 45–46, 317–320: Statements that CRD and BAT ‘enhance accuracy’ or ‘enhance sensitivity and specificity’ imply improved diagnostic performance. Given the retrospective, cross-sectional design of the study, I recommend rephrasing these statements to emphasize association or supportive clinical use rather than diagnostic enhancement.

2.Lines 68–72, 79–82: The discussion of limitations of conventional diagnostics and the potential benefits of CRD and BAT overlaps conceptually and leaves some speculations regarding improved subcutaneous immunotherapy qualification. I suggest condensing these sections and clarifying that the statements reflect hypotheses or emerging evidence rather than established clinical practice.

3.Lines 104–107: Patients in groups B and C were subdivided by reaction severity without a clear explanation of the rationale. I recommend to briefly clarify how this stratification aligns with the study objectives.

4.Lines 120–127, 321–326: BAT was conducted using a single venom concentration without dose–response testing. I recommend acknowledging this limitation explicitly in the limitations section and discussing its potential impact on interpretation of basophil sensitivity.

5.Lines 150–151: No correction for multiple testing was applied despite numerous correlation analyses. I recommend providing a clearer justification for this decision, even within an exploratory framework.

6.Lines 169–172: Missing data are reported, but their potential impact on bias or statistical power is not discussed. I suggest briefly stating whether the missing data was assumed to be at random.

Reviewer #3: This registry-based cross-sectional study analyzes the utility of the Basophil Activation Test (BAT) and Component-Resolved Diagnostics (CRD) in 154 patients evaluated for Hymenoptera venom allergy. The topic is clinically relevant, particularly regarding the specific sensitization profiles in Central Europe. (In addition, when considering the therapeutic potential of venom extracts, it can also give an insight in clinical context.) The manuscript presents interesting data regarding the correlation between specific IgE (sIgE) components and BAT reactivity.

However, the manuscript requires revision before it can be accepted. There are gaps in the reporting of methodology (specifically the BAT protocol and patient grouping criteria), discrepancies in the results (missing data in tables, conflicting statistical significance), and issues with data availability statements. Furthermore, the figures and tables require reorganization to improve readability and standard scientific formatting.

Below are my specific comments arranged by section.

#Major Revisions

1. There is a contradiction regarding data access. The metadata states "No - some restrictions will apply", yet the text under Data Availability states "All relevant data have been included within the paper and its Supporting Information files". If the data is fully available in S1 Table, the metadata should be updated to "Yes." If restrictions apply, the text must explain them.

2. The Methods section states that participants were divided into three groups (A, B, C) "according to the final diagnosis and qualification for treatment". However, the specific clinical criteria used to make these assignments are missing.

- The definition for Group A is currently located in the Introduction ("absence of confirmed venom allergy") rather than the Methods. Please provide the definition or criteria of group A, B and C to the Methods. Please define what constitutes "absence of confirmed allergy" (e.g., negative skin tests? negative sIgE?) and state the criteria (e.g., skin test reaction size, sIgE thresholds) used to categorize a patient into the Bee group vs. the Wasp group, also shown in Table 6 which separates patients based on qualification for Bee vs. Wasp immunotherapy.

3. Missing Methodological Details (BAT Protocol)** The "Laboratory procedures" section can be strengthened by adding the detail required for replication.

- In line 122 and line 128, the duration of the incubation is not stated.

- In line 128, it is unclear if staining occurred simultaneously with stimulation or as a subsequent step. Please specify the time and temperature conditions for the antibody staining.

- Was a stopping solution (e.g., EDTA) used to halt the reaction?

4. Please add the row for rVes v 5 to Table 3, which is omitted despite the text identifying it as a major component with significant correlations.

5.Figure 3 identifies a patient with double-positive reactivity. Please clarify in the Methods or Results section which group (B or C) this patient was assigned to, or if they were treated as a separate entity, as this affects the specificity analysis of the respective groups.

6. The Results section states that "nonspecific reactivity assessed by the positive control did not differ significantly" among groups. However, Table 3 reports a p-value of 0.046 for "BAT control pos.", which is statistically significant (<0.05). Please clarify this discrepancy.

7. The Conclusion section need to be improved by addressing the study objectives mentioned in the Introduction section.

- The conclusion focuses on specific molecules (Api m 1, Ves v 5) but does not explicitly relate the findings back to the differentiation of the three study groups (A, B, C) as proposed in the primary objective.

- In line 87-88, "The secondary objective was to analyze the association between the investigated parameters and the severity of anaphylactic reactions in the medical history of participants." I suggest to state even the negative finding, no association, in the Conclusion.

#Minor Revisions

1.In Methods Section,

- In "Setting and study participants", please replace "included all adult patients" with the specific number (n=154) to provide immediate context on the study power.

- There is potential confusion regarding consent. It stated "individuals unable to provide informed consent were not referred", but later says consent was "waived". Please clarify that patients lacking legal capacity for _clinical_ consent were excluded, which is distinct from the _research_ consent waiver.

- Please mention the approximate volume of blood collected to indicate the sample requirement for this battery of tests.

2.In Result Section

- I suggest clarifying the sentence ""BAT results were significantly higher for the venom of culprit insect" for general readers.

3. In Discussion Section

- In Discussion section, please remove the bulleted list of limitations and rewrite this as a continuous narrative paragraph.

- In the Discussion, the statement "few previous reports have examined..." requires citations (e.g., referencing Eberlein et al. or Korošec et al.).

4.In Figures

- I suggest to merge figure 1 and 2. These figures show the same groups responding to different stimuli. It would be much clearer to merge them into a single figure with two panels ((A): Wasp stimulation, (B) Honeybee stimulation). Ensure Y-axes have identical scales and titles.

- In figure 3, please add row labels (A, B, C, D) directly to the image and update the caption to match. The current description ("Following lines from top to bottom") is difficult to follow.

- In figure 4, please add clear axis titles (e.g., "sIgE Components", "Basophil Activation") to make the heatmap self-explanatory.

5.In tables.

- The URL citation in the title of Table 1 should be converted to a formal reference. I also suggest to consider to move this table to a more proper location not to disrupts the Introduction or I'd like to additional explanation why only eight allergen picked in this study.

- I suggest to reorganize table 2 and 3 into one with patient demographics(combining age and sex) and the other with laboratory findings(Tryptase, cells, slgE, BAT).

- 'Table 4. Distribution of missing data' disrupts the flow of the results. I suggest to merge it to the new table with laboratory findings or move it to Supplementary Information.

- The readability of Table 5 can be improved. Please regroup the columns so that **rho** and **p-value** are listed side-by-side under their respective headers (BAT Wasp / BAT Honeybee).

- All abbreviations used in tables (e.g., SD, Med, NEUT) must be defined in the table footnotes.

6. Style

- Please unify the use of abbreviation throughout the manuscript. The full name (e.g. Apis mellifera for Api m) should be defined upon first use in the body text and the abbreviation should be use consistently after it is defined (e.g. sIgE)

Reviewer #4: The manuscript entitled "Association between results of component-resolved diagnostics and basophil activation in Hymenoptera venom allergy" reports on a retrospective cross-sectional study that evaluates the association between component-resolved diagnostics (CRD) and the basophil activation test (BAT) in 154 adults with Hymenoptera venom allergy. Patients were divided into three groups: not qualified for subcutaneous immunotherapy (SCIT), qualified for bee venom SCIT, and qualified for wasp venom SCIT.

The key findings include moderate correlations between BAT results and sIgE levels to Api m 1 and Ves v 5, no association between test parameters (BAT, sIgE) and the severity of prior sting reactions (Müller grades), and that CRD and BAT may provide complementary diagnostic information, particularly in cases of polysensitization or ambiguous conventional test results.

The study is well-designed, with appropriate statistical methods and a balanced discussion of limitations. However, minor revisions are needed to clarify the study rationale and clinical implications, ensuring accessibility for PLOS ONE's broad readership.

Minor Revisions Required

1.Abstract Issue: The problem statement is underdeveloped. The rationale for comparing CRD and BAT is not explicitly stated. Please explain the interest to compare these two methods in the diagnostic and therapeutic management of HVA patients.

Refine the clinical implications: I do not think one can assert "enhances diagnostic accuracy" in the conclusion since the study objectives were not focused on diagnostic accuracy (sensitivity, specificity etc.). Instead, I would suggest replacing it with something like: CRD and BAT provide complementary information, particularly in cases of polysensitization or ambiguous conventional test results, potentially improving patient selection for SCIT.

2.Introduction Issue: Briefly explain SCIT indications before this sentence: "The combined use of BAT and CRD may potentially improve the accuracy of qualification for subcutaneous allergen immunotherapy (SCIT), etc."

The problem statement is underdeveloped. The rationale for comparing CRD and BAT is not explicitly stated. Please explain the interest to compare these two methods in the diagnostic and therapeutic management of HVA patients.

**********

6. PLOS authors have the option to publish the peer review history of their article (what does this mean?). If published, this will include your full peer review and any attached files.

If you choose “no”, your identity will remain anonymous but your review may still be made public.

**Do you want your identity to be public for this peer review?** For information about this choice, including consent withdrawal, please see our Privacy Policy.

Reviewer #1: **Yes:** Dr Daniel Elbirt

Reviewer #2: **Yes:** Mary Olorunkosebi

Reviewer #3: **Yes:** Heejin Jo

Reviewer #4: No

**********

[NOTE: If reviewer comments were submitted as an attachment file, they will be attached to this email and accessible via the submission site. Please log into your account, locate the manuscript record, and check for the action link "View Attachments". If this link does not appear, there are no attachment files.]

To ensure your figures meet our technical requirements, please review our figure guidelines: https://journals.plos.org/plosone/s/figures

You may also use PLOS’s free figure tool, NAAS, to help you prepare publication quality figures: https://journals.plos.org/plosone/s/figures#loc-tools-for-figure-preparation.

NAAS will assess whether your figures meet our technical requirements by comparing each figure against our figure specifications.

# Author comment: Author response to Decision Letter 1

**DOI:** 10.1371/journal.pone.0350189.r002

11 Mar 2026

Warsaw, March 9th, 2025

Dear Editor and Reviewers,

On behalf of my co-authors, I am glad to submit our revised manuscript entitled “Association between results of component-resolved diagnostics and basophil activation in Hymenoptera venom allergy: a registry-based cross-sectional study in adults” for publication in PLOS ONE. We thank the Reviewers for the careful assessment of our manuscript and for their precise feedback.

We would also like to provide additional information and responses to the following issues which have been raised by the Reviewers.

"2. Thank you for stating the following in the Acknowledgments Section of your manuscript:

“The authors received no specific funding for the presented work.”

We note that you have provided funding information that is not currently declared in your Funding Statement. However, funding information should not appear in the Acknowledgments section or other areas of your manuscript. We will only publish funding information present in the Funding Statement section of the online submission form.

Please remove any funding-related text from the manuscript and let us know how you would like to update your Funding Statement. Currently, your Funding Statement reads as follows:

“The author(s) received no specific funding for this work.”

Please include your amended statements within your cover letter; we will change the online submission form on your behalf."

According to the Reviewer's suggestion, we have removed funding-related text from the manuscript.

"Reviewer #2: The author has carried out a well-designed, registry-based, cross-sectional study evaluating the association between component-resolved diagnostics (CRD) and basophil activation testing (BAT) in adults with Hymenoptera venom allergy. It is clinically relevant and addresses a clear diagnostic challenge in allergy and immunology. The use of clearly defined patient groups, and integration of CRD with functional testing are notable strengths of this study. This study addresses an important diagnostic issue in venom allergy and presents clinically meaningful findings, but the author needs to address some minor clarifications in methodology, and interpretation of results.

1.Lines 45–46, 317–320: Statements that CRD and BAT ‘enhance accuracy’ or ‘enhance sensitivity and specificity’ imply improved diagnostic performance. Given the retrospective, cross-sectional design of the study, I recommend rephrasing these statements to emphasize association or supportive clinical use rather than diagnostic enhancement.

We agree and have rephrased lines 45-46 and 317-320 accordingly.

2.Lines 68–72, 79–82: The discussion of limitations of conventional diagnostics and the potential benefits of CRD and BAT overlaps conceptually and leaves some speculations regarding improved subcutaneous immunotherapy qualification. I suggest condensing these sections and clarifying that the statements reflect hypotheses or emerging evidence rather than established clinical practice.

According to the suggestion, we have condensed lines 68-72 and 79-82 in the revised version in order to improve clarity.

3.Lines 104–107: Patients in groups B and C were subdivided by reaction severity without a clear explanation of the rationale. I recommend to briefly clarify how this stratification aligns with the study objectives."

In the revised version, we have provided a brief explanation of the rationale. This was intended to enable the assessment of associations between BAT and CRD results and the severity of allergic symptoms.

"4.Lines 120–127, 321–326: BAT was conducted using a single venom concentration without dose–response testing. I recommend acknowledging this limitation explicitly in the limitations section and discussing its potential impact on interpretation of basophil sensitivity.

We agree and have provided a short explanation in the limitations section of the revised manuscript.

5.Lines 150–151: No correction for multiple testing was applied despite numerous correlation analyses. I recommend providing a clearer justification for this decision, even within an exploratory framework."

As the correlation analyses were exploratory in nature and aimed at identifying potential associations between laboratory parameters, we chose not to apply a strict correction, which could substantially increase the risk of type II error in this relatively moderate sample size. The results should therefore be interpreted as hypothesis-generating rather than confirmatory. This clarification has been added to the Methods section.

"6.Lines 169–172: Missing data are reported, but their potential impact on bias or statistical power is not discussed. I suggest briefly stating whether the missing data was assumed to be at random."

In our opinion, the proportion of missing data was low and distributed across the study groups without a discernible pattern. The missing values were primarily due to technical or logistical reasons (e.g., insufficient sample material), and were therefore assumed to be missing at random. This clarification has been added to the Discussion section.

"Reviewer #3: This registry-based cross-sectional study analyzes the utility of the Basophil Activation Test (BAT) and Component-Resolved Diagnostics (CRD) in 154 patients

evaluated for Hymenoptera venom allergy. The topic is clinically relevant, particularly regarding the specific sensitization profiles in Central Europe. (In addition, when considering the therapeutic potential of venom extracts, it can also give an insight in clinical context.) The manuscript presents interesting data regarding the correlation between specific IgE (sIgE) components and BAT reactivity. However, the manuscript requires revision before it can be accepted. There are gaps in the reporting of methodology (specifically the BAT protocol and patient grouping criteria), discrepancies in the results (missing data in tables, conflicting statistical significance), and issues with data availability statements. Furthermore, the figures and tables require reorganization to improve readability and standard scientific formatting. Below are my specific comments arranged by section.

#Major Revisions 1. There is a contradiction regarding data access. The metadata states "No - some restrictions will apply", yet the text under Data Availability states "All relevant data have been included within the paper and its Supporting Information files". If the data is fully available in S1 Table, the metadata should be updated to "Yes." If restrictions apply, the text must explain them.

We have revised the metadata in order to avoid this contradiction.

2. The Methods section states that participants were divided into three groups (A, B, C) "according to the final diagnosis and qualification for treatment". However, the specific clinical criteria used to make these assignments are missing. - The definition for Group A is currently located in the Introduction ("absence of confirmed venom allergy") rather than the Methods. Please provide the definition or criteria of group A, B and C to the Methods. Please define what constitutes "absence of confirmed allergy" (e.g., negative skin tests? negative sIgE?) and state the criteria (e.g., skin test reaction size, sIgE thresholds) used to categorize a patient into the Bee group vs. the Wasp group, also shown in Table 6 which separates patients based on qualification for Bee vs. Wasp immunotherapy."

Group A consisted of patients who were not diagnosed with venom allergy, defined as the absence of venom-specific IgE antibodies and a negative basophil activation test. The threshold for sIgE was 0.35 IU/mL. Group B consisted of patients who were qualified for bee venom immunotherapy, and Group C consisted of patients who were qualified for wasp venom immunotherapy, respectively. The aforementioned information has been incorporated into the revised version of the manuscript.

"3. Missing Methodological Details (BAT Protocol)** The "Laboratory procedures" section can be strengthened by adding the detail required for replication. - In line 122 and line 128, the duration of the incubation is not stated. - In line 128, it is unclear if staining occurred simultaneously with stimulation or as a subsequent step. Please specify the time and temperature conditions for the antibody staining. - Was a stopping solution (e.g., EDTA) used to halt the reaction?"

We have improved the “Laboratory procedures” section profoundly according to the advice. The incubation consisted of two steps: 25 minutes in an air incubator, followed by 20 minutes at 2–8 °C. Staining was performed as a subsequent step after stimulation. Twenty μL of staining reagent was added to each tube. Samples were then incubated for 20 minutes at 2–8 °C. No stopping solution was used. Immediately after the staining step, a lysing solution was added. After 5 minutes—once red blood cells were lysed—the samples were centrifuged, the supernatant was removed, and the pellet was resuspended in 200 μL of PBS. The samples were then analyzed immediately using a flow cytometer.

"4. Please add the row for rVes v 5 to Table 3, which is omitted despite the text identifying it as a major component with significant correlations."

We have added the missing row in the revised version.

"5.Figure 3 identifies a patient with double-positive reactivity. Please clarify in the Methods or Results section which group (B or C) this patient was assigned to, or if they were treated as a separate entity, as this affects the specificity analysis of the respective groups."

This result is presented mainly as a didactic example of an unusual phenomenon. The patient referred to in this comment was classified into Group B, as wasp venom allergy was considered the primary cause of anaphylaxis in this case and constituted the target of immunotherapy. We have provided additional clarification in the Results section.

"6. The Results section states that "nonspecific reactivity assessed by the positive control did not differ significantly" among groups. However, Table 3 reports a p-value of 0.046 for "BAT control pos.", which is statistically significant (<0.05). Please clarify this discrepancy."

While the overall ANOVA/Kruskal–Wallis test showed a p-value of 0.046 for the BAT positive control, none of the post hoc pairwise comparisons reached statistical significance. Therefore, although the global test suggested a borderline difference, no significant differences were identified between specific groups. To avoid confusion, we have revised the Results section to reflect this more precisely.

"7. The Conclusion section need to be improved by addressing the study objectives mentioned in the Introduction section. - The conclusion focuses on specific molecules (Api m 1, Ves v 5) but does not explicitly relate the findings back to the differentiation of the three study groups (A, B, C) as proposed in the primary objective. - In line 87-88, "The secondary objective was to analyze the association between the investigated parameters and the severity of anaphylactic reactions in the medical history of participants." I suggest to state even the negative finding, no association, in the Conclusion."

The Conclusion has been reformulated to better align with the objectives stated in the Introduction, including both the group differentiation analysis and the negative findings regarding reaction severity.

"#Minor Revisions 1.In Methods Section, - In "Setting and study participants", please replace "included all adult patients" with the specific number (n=154) to provide immediate context on the study power. - There is potential confusion regarding consent. It stated "individuals unable to provide informed consent were not referred", but later says consent was "waived". Please clarify that patients lacking legal capacity for _clinical_ consent were excluded, which is distinct from the _research_ consent waiver. - Please mention the

approximate volume of blood collected to indicate the sample requirement for this battery of tests."

We have implemented changes according to the suggestions. Approximately 10–15 mL of peripheral venous blood was collected from each participant to perform all laboratory analyses.

"2.In Result Section - I suggest clarifying the sentence ""BAT results were significantly higher for the venom of culprit insect" for general readers."

We have changed “BAT results” to “basophil reactivity” in order to clarify the sentence.

"3. In Discussion Section - In Discussion section, please remove the bulleted list of limitations and rewrite this as a continuous narrative paragraph. - In the Discussion, the statement "few previous reports have examined..." requires citations (e.g., referencing Eberlein et al. or Korošec et al.)."

The Discussion has been rewritten in accordance with the Reviewer’s advice. We have added suggested citations.

"4.In Figures - I suggest to merge figure 1 and 2. These figures show the same groups responding to different stimuli. It would be much clearer to merge them into a single figure with two panels ((A): Wasp stimulation, (B) Honeybee stimulation). Ensure Y-axes have identical scales and titles. - In figure 3, please add row labels (A, B, C, D) directly to the image and update the caption to match. The current description ("Following lines from top to bottom") is difficult to follow. - In figure 4, please add clear axis titles (e.g., "sIgE Components", "Basophil Activation") to make the heatmap self-explanatory."

Figures 1 and 2 have been merged into a single figure with two panels. The row labels have been added to Figure 3, and the captions have been updated. We have also provided additional axis titles.

"5.In tables. - The URL citation in the title of Table 1 should be converted to a formal reference. I also suggest to consider to move this table to a more proper location not to disrupts the Introduction or I'd like to additional explanation why only eight allergen picked in this study. - I suggest to reorganize table 2 and 3 into one with patient demographics(combining age and sex) and the other with laboratory findings(Tryptase, cells, slgE, BAT). - 'Table 4. Distribution of missing data' disrupts the flow of the results. I suggest to merge it to the new table with laboratory findings or move it to Supplementary Information. - The readability of Table 5 can be improved. Please regroup the columns so that **rho** and **p-value** are listed side-by-side under their respective headers (BAT Wasp / BAT Honeybee). - All abbreviations used in tables (e.g., SD, Med, NEUT) must be defined in the table footnotes."

We have converted the URL citation to a formal reference. We have also moved the aforementioned table to the Methods section. Additional explanation regarding the chosen allergens has been provided in the revised version of the manuscript. According to the suggestion, we have merged Table 2 and Table 3 into one, and we have also transferred Table 4 to the Supporting Information. The columns of Table 5 have been regrouped for clarity. Missing abbreviations have been defined in the table footnotes.

"6. Style - Please unify the use of abbreviation throughout the manuscript. The full name (e.g. Apis mellifera for Api m) should be defined upon first use in the body text and the abbreviation should be use consistently after it is defined (e.g. sIgE)"

We have unified abbreviations throughout the revised manuscript and have added missing full names.

"Reviewer #4: The manuscript entitled "Association between results of component-resolved diagnostics and basophil activation in Hymenoptera venom allergy" reports on a retrospective cross-sectional study that evaluates the association between component-resolved diagnostics (CRD) and the basophil activation test (BAT) in 154 adults with Hymenoptera venom allergy. Patients were divided into three groups: not qualified for subcutaneous immunotherapy (SCIT), qualified for bee venom SCIT, and qualified for wasp venom SCIT. The key findings include moderate correlations between BAT results and sIgE levels to Api m 1 and Ves v 5, no association between test parameters (BAT, sIgE) and the severity of prior sting reactions (Müller grades), and that CRD and BAT m

Attachment Submitted filename: Response to Reviewers.pdf

# Editor report: Decision Letter 1

**DOI:** 10.1371/journal.pone.0350189.r003

**Authors:** Petr Heneberg

12 Mar 2026

PONE-D-25-68655R1

Association between results of component-resolved diagnostics and basophil activation in Hymenoptera venom allergy: a registry-based cross-sectional study in adults.

PLOS One

Dear Dr. Piwowarek,

Thank you for submitting your manuscript to PLOS ONE. After careful consideration, we feel that it has merit but does not fully meet PLOS ONE’s publication criteria as it currently stands. Therefore, we invite you to submit a revised version of the manuscript that addresses the points raised during the review process.

Please submit your revised manuscript by Apr 26 2026 11:59PM. If you will need more time than this to complete your revisions, please reply to this message or contact the journal office at plosone@plos.org. When you're ready to submit your revision, log on to https://www.editorialmanager.com/pone/ and select the 'Submissions Needing Revision' folder to locate your manuscript file.

Please include the following items when submitting your revised manuscript:

- A letter that responds to each point raised by the academic editor and reviewer(s). You should upload this letter as a separate file labeled 'Response to Reviewers'.
- A marked-up copy of your manuscript that highlights changes made to the original version. You should upload this as a separate file labeled 'Revised Manuscript with Track Changes'.
- An unmarked version of your revised paper without tracked changes. You should upload this as a separate file labeled 'Manuscript'.

-->If you would like to make changes to your financial disclosure, please include your updated statement in your cover letter. Guidelines for resubmitting your figure files are available below the reviewer comments at the end of this letter.

If applicable, we recommend that you deposit your laboratory protocols in protocols.io to enhance the reproducibility of your results. Protocols.io assigns your protocol its own identifier (DOI) so that it can be cited independently in the future. For instructions see: https://journals.plos.org/plosone/s/submission-guidelines#loc-laboratory-protocols. Additionally, PLOS ONE offers an option for publishing peer-reviewed Lab Protocol articles, which describe protocols hosted on protocols.io. Read more information on sharing protocols at https://plos.org/protocols?utm_medium=editorial-email&utm_source=authorletters&utm_campaign=protocols.

We look forward to receiving your revised manuscript.

Kind regards,

Petr Heneberg

Academic Editor

PLOS One

Journal Requirements:

If the reviewer comments include a recommendation to cite specific previously published works, please review and evaluate these publications to determine whether they are relevant and should be cited. There is no requirement to cite these works unless the editor has indicated otherwise.

Please review your reference list to ensure that it is complete and correct. If you have cited papers that have been retracted, please include the rationale for doing so in the manuscript text, or remove these references and replace them with relevant current references. Any changes to the reference list should be mentioned in the rebuttal letter that accompanies your revised manuscript. If you need to cite a retracted article, indicate the article’s retracted status in the References list and also include a citation and full reference for the retraction notice.

Additional Editor Comments:

In my view, the manuscript still attributes more certainty to the results than the design permits. A retrospective cross-sectional study can show that certain markers are associated with BAT results in this sample, but it cannot establish that one marker exerts the strongest impact on diagnosis or that it has a decisive biological role. Expressions such as influence, impact, and decisive role suggest causal hierarchy or clinical superiority. The data presented support a more limited statement, namely that Api m 1 and Ves v 5 were the markers most strongly correlated with BAT among the variables examined.

The way the groups are defined leaves unresolved circularity. Group A is characterized in part by negative venom-specific IgE and negative BAT, yet the manuscript does not specify in operational terms how patients were assigned to the bee and wasp immunotherapy groups. If clinical history, skin testing, extract-specific IgE, BAT, or component-resolved diagnostics contributed to assignment, and those same variables are then compared across groups as if they were independent outcomes, the observed differences may largely reproduce the initial classification logic. The manuscript should therefore provide a stepwise diagnostic algorithm showing which criteria determined group membership and which measures were reserved for comparative analysis.

The ethics wording remains difficult to reconcile. The text states that individuals unable to provide informed consent were excluded, but also states that written informed consent was waived because the study was retrospective and anonymized. As written, it is not clear whether the exclusion referred to inability to consent to routine clinical procedures, inability to consent to research participation, or something else. There is also a chronology issue that should be addressed explicitly, since the patient evaluation period ended before the stated ethics approval date. If the approval covered retrospective analysis of already collected data, the manuscript should say that directly and unambiguously.

The statistical approach is not sufficiently guarded in relation to the number of analyses performed. The manuscript labels the work exploratory, but it still interprets multiple nominal p values below 0.05 as positive findings across a broad set of tests and correlations. In that setting, isolated borderline results can arise from multiplicity alone. Several of the reported associations fall very close to the conventional threshold, which limits confidence in their stability. A stricter presentation would either apply a multiple-testing correction or state clearly that these p values are descriptive and hypothesis-generating rather than confirmatory.

Some reported summary statistics appear inconsistent enough to require a full audit of the tables. The neutrophil data are one example, where the means are presented in the thousands but the standard deviations are around 1.26 and 1.27, which suggests a unit mismatch, formatting error, or incorrect transcription. Another problematic entry is the severe bee subgroup value for rApi m 3, where the reported mean, standard deviation, and median do not appear to form a plausible combination. These inconsistencies should be checked against the raw dataset and the statistical output, with corrections made wherever necessary.

The mechanistic explanation offered for Api m 1 and Ves v 5 is not well grounded in the underlying biology. The manuscript suggests that their relatively low molecular weight may increase accessibility to FcεRI receptors, but FcεRI binds IgE already attached to the basophil surface, not the allergen itself. Basophil activation depends on allergen-mediated cross-linking of receptor-bound IgE, and that process is shaped by factors such as sensitization profile, epitope architecture, and functional binding characteristics. The proposed explanation is therefore speculative and should not be presented as if it were a supported interpretation of the current results.

The severity analysis should be stated more carefully. The absence of a statistically significant association in these subgroups does not justify a firm conclusion that severity is unrelated to the measured markers. The bee subgroup is especially limited because the mild-reaction category contains very few patients compared with the severe category, and that imbalance reduces the ability to detect anything other than large differences. The manuscript should therefore report that no association with severity was detected in this dataset, rather than presenting the result as evidence against any relationship.

In my view, the manuscript was improved when compared to its first version, but the above-mentioned issues need to be addressed in order to avoid data overinterpretation and misinterpretation.

[Note: HTML markup is below. Please do not edit.]

[NOTE: If reviewer comments were submitted as an attachment file, they will be attached to this email and accessible via the submission site. Please log into your account, locate the manuscript record, and check for the action link "View Attachments". If this link does not appear, there are no attachment files.]

To ensure your figures meet our technical requirements, please review our figure guidelines: https://journals.plos.org/plosone/s/figures

You may also use PLOS’s free figure tool, NAAS, to help you prepare publication quality figures: https://journals.plos.org/plosone/s/figures#loc-tools-for-figure-preparation.

NAAS will assess whether your figures meet our technical requirements by comparing each figure against our figure specifications.

# Author comment: Author response to Decision Letter 2

**DOI:** 10.1371/journal.pone.0350189.r004

30 Mar 2026

Dear Editor and Reviewers,

On behalf of all co-authors, I am glad to resubmit our revised manuscript entitled “Association between results of component-resolved diagnostics and basophil activation in Hymenoptera venom allergy: a registry-based cross-sectional study in adults” for publication in PLOS ONE. We thank the Reviewer for the meticulous assessment of our manuscript and for the precise feedback, which motivated us to improve our work.

We would also like to provide additional information and necessary responses to the following issues which have been raised by the Reviewer.

"In my view, the manuscript still attributes more certainty to the results than the design permits. A retrospective cross-sectional study can show that certain markers are associated with BAT results in this sample, but it cannot establish that one marker exerts the strongest impact on diagnosis or that it has a decisive biological role. Expressions such as influence, impact, and decisive role suggest causal hierarchy or clinical superiority. The data presented support a more limited statement, namely that Api m 1 and Ves v 5 were the markers most strongly correlated with BAT among the variables examined."

We agree that the retrospective cross-sectional design of our study does not allow for establishing hierarchical or decisive roles of individual markers. In response, we have carefully revised the manuscript throughout to avoid language suggesting causality or clinical superiority. Specifically, terms such as “impact,” “influence,” and “decisive role” have been removed or replaced with more appropriate expressions such as “correlation”. These changes have been implemented across the Abstract, Results, and Discussion sections to ensure consistency with the study design and to avoid overinterpretation.

"The way the groups are defined leaves unresolved circularity. Group A is characterized in part by negative venom-specific IgE and negative BAT, yet the manuscript does not specify in operational terms how patients were assigned to the bee and wasp immunotherapy groups. If clinical history, skin testing, extract-specific IgE, BAT, or component-resolved diagnostics contributed to assignment, and those same variables are then compared across groups as if they were independent outcomes, the observed differences may largely reproduce the initial classification logic. The manuscript should therefore provide a stepwise diagnostic algorithm showing which criteria determined group membership and which measures were reserved for comparative analysis."

We thank the Editor for this important methodological comment. We agree that the original version of the manuscript did not sufficiently clarify how patients were assigned to study groups and how this relates to the variables analyzed, which could raise concerns about circularity.

In response, we have revised the Methods section of manuscript to include a detailed stepwise diagnostic algorithm describing:

• the role of clinical history and serum venom extract-specific IgE in the initial assessment of sensitization

• the criteria used for qualification to bee or wasp venom immunotherapy

• the role of component-resolved diagnostics (CRD) and the basophil activation test (BAT) as complementary investigations

In addition, we have addressed this issue in the Limitations section, where we explicitly acknowledge that some of the diagnostic measures analyzed, particularly CRD, contributed to routine clinical decision-making. Therefore, between-group comparisons are presented as descriptive, and some degree of circularity cannot be excluded. These revisions were made to ensure transparent reporting of the diagnostic workflow and to avoid overinterpretation of between-group differences.

"The ethics wording remains difficult to reconcile. The text states that individuals unable to provide informed consent were excluded, but also states that written informed consent was waived because the study was retrospective and anonymized. As written, it is not clear whether the exclusion referred to inability to consent to routine clinical procedures, inability to consent to research participation, or something else. There is also a chronology issue that should be addressed explicitly, since the patient evaluation period ended before the stated ethics approval date. If the approval covered retrospective analysis of already collected data, the manuscript should say that directly and unambiguously."

In order to avoid unambiguity, we have revised both the Ethics statement and the Methods section to ensure consistency and transparency. We have clarified that the study represents a retrospective analysis of previously collected clinical data, and that the Bioethics Committee approval covered this retrospective use of anonymized data. We have revised the exclusion criteria to clearly indicate that they refer to the inability to provide informed consent to routine clinical diagnostic procedures or venom immunotherapy, rather than to research participation. We have highlighted difference between the patient evaluation period and the later data extraction and analysis phase, which took place after ethics approval.

"The statistical approach is not sufficiently guarded in relation to the number of analyses performed. The manuscript labels the work exploratory, but it still interprets multiple nominal p values below 0.05 as positive findings across a broad set of tests and correlations. In that setting, isolated borderline results can arise from multiplicity alone. Several of the reported associations fall very close to the conventional threshold, which limits confidence in their stability. A stricter presentation would either apply a multiple-testing correction or state clearly that these p values are descriptive and hypothesis-generating rather than confirmatory."

We agree with the Editor that, given the number of analyses performed, the results should be interpreted with appropriate caution. To address this, we have revised the manuscript to explicitly state that:

• the analyses are exploratory in nature

• the reported p-values are descriptive and hypothesis-generating rather than confirmatory

• findings, particularly those with p-values close to the conventional threshold, should be interpreted cautiously in the context of multiple comparisons

We have not applied formal multiple-testing correction, as the primary aim of the study was exploratory; however, this limitation is now clearly acknowledged and reflected in the Methods section.

"Some reported summary statistics appear inconsistent enough to require a full audit of the tables. The neutrophil data are one example, where the means are presented in the thousands but the standard deviations are around 1.26 and 1.27, which suggests a unit mismatch, formatting error, or incorrect transcription. Another problematic entry is the severe bee subgroup value for rApi m 3, where the reported mean, standard deviation, and median do not appear to form a plausible combination. These inconsistencies should be checked against the raw dataset and the statistical output, with corrections made wherever necessary."

We thank the Editor for carefully identifying these inconsistencies. We have performed an additional audit of the dataset, statistical outputs, and reported summary statistics.

This review revealed two issues:

1. Neutrophil counts:

In some table cells, neutrophil values were inadvertently reported using different unit conventions (absolute counts vs. values expressed in thousands), which resulted in impossible combinations of means and standard deviations. All neutrophil data have now been standardized to a single, consistent unit.

2. rApi m 3 values (severe anaphylaxis honeybee subgroup):

The previously reported standard deviation and median were mistakenly transposed. This error has now been corrected based on the original statistical output.

We regret these errors and appreciate the opportunity to correct them. These changes do not materially affect the overall results or conclusions of the study.

"The mechanistic explanation offered for Api m 1 and Ves v 5 is not well grounded in the underlying biology. The manuscript suggests that their relatively low molecular weight may increase accessibility to FcεRI receptors, but FcεRI binds IgE already attached to the basophil surface, not the allergen itself. Basophil activation depends on allergen-mediated cross-linking of receptor-bound IgE, and that process is shaped by factors such as sensitization profile, epitope architecture, and functional binding characteristics. The proposed explanation is therefore speculative and should not be presented as if it were a supported interpretation of the current results."

We agree with the concerns raised regarding the mechanistic interpretation. In response, we have removed the speculative explanation suggesting that the relatively low molecular weight of Api m 1 and Ves v 5 may increase accessibility to FcεRI receptors. We agree that this interpretation was not adequately supported by current knowledge of basophil activation mechanisms. The revised manuscript no longer includes this statement.

"The severity analysis should be stated more carefully. The absence of a statistically significant association in these subgroups does not justify a firm conclusion that severity is unrelated to the measured markers. The bee subgroup is especially limited because the mild-reaction category contains very few patients compared with the severe category, and that imbalance reduces the ability to detect anything other than large differences. The manuscript should therefore report that no association with severity was detected in this dataset, rather than presenting the result as evidence against any relationship."

We agree that the interpretation of the severity analysis should be more cautious. In response, we have revised the manuscript to avoid any statements suggesting the firm absence of a relationship between the investigated markers and reaction severity. The Discussion and Conclusions sections have been updated to state that no statistically significant association was detected in this dataset, rather than implying a lack of association. We have also emphasized the limited size of certain subgroups, particularly the honeybee subgroup with a small number of patients in the mild-reaction category, which reduces the ability to detect anything other than large differences.

We respectfully suggest that our manuscript fits well within the scope of PLOS ONE, which values scientifically rigorous, methodologically sound studies that advance knowledge across disciplines and are relevant for clinical and translational practice.

Thank you for considering our work. We are looking forward to your feedback.

Sincerely,

Krzysztof Piwowarek, MD, PhD

Department of Internal Medicine, Infectious Diseases and Allergology

Military Institute of Medicine – National Research Institute

Warsaw, Poland

Attachment Submitted filename: Response to Reviewers.docx

# Editor report: Decision Letter 2

**DOI:** 10.1371/journal.pone.0350189.r005

**Authors:** Petr Heneberg

31 Mar 2026

PONE-D-25-68655R2

Association between results of component-resolved diagnostics and basophil activation in Hymenoptera venom allergy: a registry-based cross-sectional study in adults.

PLOS One

Dear Dr. Piwowarek,

Thank you for submitting your manuscript to PLOS ONE. After careful consideration, we feel that it has merit but does not fully meet PLOS ONE’s publication criteria as it currently stands. Therefore, we invite you to submit a revised version of the manuscript that addresses the points raised during the review process.

Please submit your revised manuscript by May 15 2026 11:59PM. If you will need more time than this to complete your revisions, please reply to this message or contact the journal office at plosone@plos.org. When you're ready to submit your revision, log on to https://www.editorialmanager.com/pone/ and select the 'Submissions Needing Revision' folder to locate your manuscript file.

Please include the following items when submitting your revised manuscript:

- A letter that responds to each point raised by the academic editor and reviewer(s). You should upload this letter as a separate file labeled 'Response to Reviewers'.
- A marked-up copy of your manuscript that highlights changes made to the original version. You should upload this as a separate file labeled 'Revised Manuscript with Track Changes'.
- An unmarked version of your revised paper without tracked changes. You should upload this as a separate file labeled 'Manuscript'.

If you would like to make changes to your financial disclosure, please include your updated statement in your cover letter. Guidelines for resubmitting your figure files are available below the reviewer comments at the end of this letter.

If applicable, we recommend that you deposit your laboratory protocols in protocols.io to enhance the reproducibility of your results. Protocols.io assigns your protocol its own identifier (DOI) so that it can be cited independently in the future. For instructions see: https://journals.plos.org/plosone/s/submission-guidelines#loc-laboratory-protocols. Additionally, PLOS ONE offers an option for publishing peer-reviewed Lab Protocol articles, which describe protocols hosted on protocols.io. Read more information on sharing protocols at https://plos.org/protocols?utm_medium=editorial-email&utm_source=authorletters&utm_campaign=protocols.

As the corresponding author, your ORCID iD is verified in the submission system and will appear in the published article. PLOS supports the use of ORCID, and we encourage all coauthors to register for an ORCID iD and use it as well. Please encourage your coauthors to verify their ORCID iD within the submission system before final acceptance, as unverified ORCID iDs will not appear in the published article. *Only* the individual author can complete the verification step; PLOS staff *cannot* verify ORCID iDs on behalf of authors.

We look forward to receiving your revised manuscript.

Kind regards,

Petr Heneberg

Academic Editor

PLOS One

Journal Requirements:

If the reviewer comments include a recommendation to cite specific previously published works, please review and evaluate these publications to determine whether they are relevant and should be cited. There is no requirement to cite these works unless the editor has indicated otherwise.

Please review your reference list to ensure that it is complete and correct. If you have cited papers that have been retracted, please include the rationale for doing so in the manuscript text, or remove these references and replace them with relevant current references. Any changes to the reference list should be mentioned in the rebuttal letter that accompanies your revised manuscript. If you need to cite a retracted article, indicate the article’s retracted status in the References list and also include a citation and full reference for the retraction notice.

Additional Editor Comments (if provided):

The manuscript was improved and the revisions were made in a correct direction, but several of them were incomplete. In my view, the main remaining concerns are the ones that still limit interpretation even after revision, together with practical fixes that could be implemented without additional wet lab work.

The severity analysis remains a key weakness. The authors now acknowledge that some subgroups were small, especially the honeybee mild-reaction subgroup, and that the findings therefore do not justify a firm conclusion. That is an improvement, but the analysis is still underpowered for a confident statement about severity-related associations. I would therefore encourage the authors to tone this down even further in the abstract, discussion, and conclusions, and to present the severity results explicitly as inconclusive subgroup analyses rather than negative findings. It would also strengthen the manuscript if the authors reported effect sizes and confidence intervals, not only p values, so that readers can better judge the uncertainty around these estimates.

Circularity remains a substantive methodological limitation. The authors added a clearer diagnostic algorithm and now acknowledge that some circularity cannot be excluded because component-resolved diagnostics contributed to routine clinical decision-making. This improves transparency, but it does not remove the issue. I would encourage the authors to distinguish more clearly which variables were used for clinical classification and which were treated as analytic outcomes, and then to frame the between-group comparisons as descriptive only. If possible, the authors could also add a sensitivity analysis excluding variables most directly involved in treatment assignment, or at least provide a table that identifies classification-related versus evaluation-only variables.

The statistical analysis part is still not very strong. The revision presents the findings more cautiously, but the manuscript still relies on multiple comparisons and nominal significance testing. A practical improvement, without requiring new experiments, would be to restructure the Results around effect sizes, confidence intervals, and a smaller set of prespecified primary comparisons, while moving more exploratory correlations to the Supplement. Alternatively, if the authors prefer to retain the present structure, the manuscript should state even more explicitly that these analyses are hypothesis-generating rather than confirmatory.

The retrospective single-center design remains an important limitation for generalizability. The authors now acknowledge this more clearly, which is appropriate, but the claims should remain tightly aligned with what this dataset can actually support. I would therefore suggest that the authors narrow the wording where needed and avoid implying broader diagnostic applicability beyond similar referral populations.

The absence of skin test data and the use of only a single BAT concentration still weaken the diagnostic interpretation. Since this cannot be remedied experimentally at this stage, I would encourage the authors to address it more explicitly in the manuscript by clarifying how these limitations constrain interpretation, and by avoiding any implication that BAT performance or thresholds were comprehensively characterized here. A brief supplementary flowchart or summary table showing which diagnostic modalities were available for how many patients would also improve transparency.

Missing data remain a lesser but still relevant concern. The explanation provided by the authors is reasonable, but the manuscript would be stronger if the authors added a compact supplementary table showing missingness by variable and group, together with a brief statement on whether complete-case analyses differed materially from the full analytic sample.

Some conclusions also still extend slightly beyond what the design can firmly support. I would encourage the authors to make the final take-home message as precise as possible, emphasizing association and complementary diagnostic information rather than clinical utility in a stronger sense. Statements about decision support would be better framed as requiring prospective validation.

[Note: HTML markup is below. Please do not edit.]

[NOTE: If reviewer comments were submitted as an attachment file, they will be attached to this email and accessible via the submission site. Please log into your account, locate the manuscript record, and check for the action link "View Attachments". If this link does not appear, there are no attachment files.]

To ensure your figures meet our technical requirements, please review our figure guidelines: https://journals.plos.org/plosone/s/figures

You may also use PLOS’s free figure tool, NAAS, to help you prepare publication quality figures: https://journals.plos.org/plosone/s/figures#loc-tools-for-figure-preparation.

NAAS will assess whether your figures meet our technical requirements by comparing each figure against our figure specifications.

# Author comment: Author response to Decision Letter 3

**DOI:** 10.1371/journal.pone.0350189.r006

8 May 2026

Warsaw, May 7th, 2026

Dear Editor and Reviewers,

On behalf of all co-authors, I am glad to resubmit our revised manuscript entitled “Association between results of component-resolved diagnostics and basophil activation in Hymenoptera venom allergy: a registry-based cross-sectional study in adults” for publication in PLOS ONE. We thank the Reviewer for the profound assessment of our manuscript and for the feedback, which has improved our work.

We would also like to provide necessary information and responses to the following issues which have been raised by the Reviewer.

“The manuscript was improved and the revisions were made in a correct direction, but several of them were incomplete. In my view, the main remaining concerns are the ones that still limit interpretation even after revision, together with practical fixes that could be implemented without additional wet lab work.

The severity analysis remains a key weakness. The authors now acknowledge that some subgroups were small, especially the honeybee mild-reaction subgroup, and that the findings therefore do not justify a firm conclusion. That is an improvement, but the analysis is still underpowered for a confident statement about severity-related associations. I would therefore encourage the authors to tone this down even further in the abstract, discussion, and conclusions, and to present the severity results explicitly as inconclusive subgroup analyses rather than negative findings. It would also strengthen the manuscript if the authors reported effect sizes and confidence intervals, not only p values, so that readers can better judge the uncertainty around these estimates.”

We agree that the severity analysis is underpowered, particularly in the honeybee mild-reaction subgroup, and does not allow for firm conclusions. In response, we have further revised the Abstract, Discussion, and Conclusions to explicitly present these results as inconclusive subgroup analyses. Additionally, we have now reported 95% confidence intervals for all severity-related analyses to better reflect the magnitude and uncertainty of the estimates. We believe these changes improve the transparency and interpretability of the results.

“Circularity remains a substantive methodological limitation. The authors added a clearer diagnostic algorithm and now acknowledge that some circularity cannot be excluded because component-resolved diagnostics contributed to routine clinical decision-making. This improves transparency, but it does not remove the issue. I would encourage the authors to distinguish more clearly which variables were used for clinical classification and which were treated as analytic outcomes, and then to frame the between-group comparisons as descriptive only. If possible, the authors could also add a sensitivity analysis excluding variables most directly involved in treatment assignment, or at least provide a table that identifies classification-related versus evaluation-only variables.”

To improve transparency and address potential circularity, we have now clearly distinguished variables used for clinical classification from those treated as analytic outcomes. This distinction has been summarized in a new table (Table 5), where variables are categorized as classification-related or evaluation-only.

“The statistical analysis part is still not very strong. The revision presents the findings more cautiously, but the manuscript still relies on multiple comparisons and nominal significance testing. A practical improvement, without requiring new experiments, would be to restructure the Results around effect sizes, confidence intervals, and a smaller set of prespecified primary comparisons, while moving more exploratory correlations to the Supplement. Alternatively, if the authors prefer to retain the present structure, the manuscript should state even more explicitly that these analyses are hypothesis-generating rather than confirmatory."

We agree and in addition to revising the statistical analysis section, we have now explicitly emphasized in the Discussion that the aforementioned analyses are exploratory and hypothesis-generating rather than confirmatory.

“The retrospective single-center design remains an important limitation for generalizability. The authors now acknowledge this more clearly, which is appropriate, but the claims should remain tightly aligned with what this dataset can actually support. I would therefore suggest that the authors narrow the wording where needed and avoid implying broader diagnostic applicability beyond similar referral populations."

We agree that the retrospective single-center design limits the generalizability of our findings. In response, we have further revised the manuscript to ensure that the conclusions remain closely aligned with the scope of the dataset and do not imply broader diagnostic applicability beyond similar referral populations.

“The absence of skin test data and the use of only a single BAT concentration still weaken the diagnostic interpretation. Since this cannot be remedied experimentally at this stage, I would encourage the authors to address it more explicitly in the manuscript by clarifying how these limitations constrain interpretation, and by avoiding any implication that BAT performance or thresholds were comprehensively characterized here. A brief supplementary flowchart or summary table showing which diagnostic modalities were available for how many patients would also improve transparency.”

We agree that the absence of skin test data and the use of a single BAT concentration limit the diagnostic interpretation of our findings. In response, we have further clarified these limitations in the Discussion, explicitly stating how they constrain interpretation and avoiding any implication that BAT performance or diagnostic thresholds were comprehensively characterized. We would also like to notice that we have already included a supplementary file summarizing data completeness, which details the availability of diagnostic modalities across the study population.

“Missing data remain a lesser but still relevant concern. The explanation provided by the authors is reasonable, but the manuscript would be stronger if the authors added a compact supplementary table showing missingness by variable and group, together with a brief statement on whether complete-case analyses differed materially from the full analytic sample.”

We have already included a supplementary table (S3 Table) summarizing missing data by variable and group, which provides an overview of data availability across the study population. In addition, we have included a brief statement in the revised manuscript addressing whether complete-case analyses differed materially from the full analytic sample.

“Some conclusions also still extend slightly beyond what the design can firmly support. I would encourage the authors to make the final take-home message as precise as possible, emphasizing association and complementary diagnostic information rather than clinical utility in a stronger sense. Statements about decision support would be better framed as requiring prospective validation.”

We agree that the conclusions should remain closely aligned with the scope of the study design. In response, we have revised the Conclusions section to ensure a more precise and cautious message, emphasizing associations and complementary diagnostic information as well as excluding broader clinical utility. We believe these changes improve the accuracy and balance of the manuscript.

We respectfully suggest that our manuscript fits within the scope of PLOS ONE, which values methodologically sound studies that advance knowledge and are relevant for clinical and translational practice.

Thank you for considering our work. We are looking forward to your feedback.

Sincerely,

Krzysztof Piwowarek, MD, PhD

Department of Internal Medicine, Infectious Diseases and Allergology

Military Institute of Medicine – National Research Institute

Warsaw, Poland

Attachment Submitted filename: Response_to_Reviewers_auresp_3.docx

# Editor report: Decision Letter 3

**DOI:** 10.1371/journal.pone.0350189.r007

**Authors:** Petr Heneberg

12 May 2026

Association between results of component-resolved diagnostics and basophil activation in Hymenoptera venom allergy: a registry-based cross-sectional study in adults.

PONE-D-25-68655R3

Dear Dr. Piwowarek,

We’re pleased to inform you that your manuscript has been judged scientifically suitable for publication and will be formally accepted for publication once it meets all outstanding technical requirements.

Within one week, you’ll receive an e-mail detailing the required amendments. When these have been addressed, you’ll receive a formal acceptance letter and your manuscript will be scheduled for publication.

An invoice will be generated when your article is formally accepted. Please note, if your institution has a publishing partnership with PLOS and your article meets the relevant criteria, all or part of your publication costs will be covered. Please make sure your user information is up-to-date by logging into Editorial Manager at Editorial Manager® and clicking the ‘Update My Information' link at the top of the page. For questions related to billing, please contact billing support.

If your institution or institutions have a press office, please notify them about your upcoming paper to help maximize its impact. If they’ll be preparing press materials, please inform our press team as soon as possible -- no later than 48 hours after receiving the formal acceptance. Your manuscript will remain under strict press embargo until 2 pm Eastern Time on the date of publication. For more information, please contact onepress@plos.org.

Kind regards,

Petr Heneberg

Academic Editor

PLOS One

Additional Editor Comments (optional):

Reviewers' comments:

# Editor report: Acceptance letter

**DOI:** 10.1371/journal.pone.0350189.r008

**Authors:** Petr Heneberg

PONE-D-25-68655R3

PLOS One

Dear Dr. Piwowarek,

I'm pleased to inform you that your manuscript has been deemed suitable for publication in PLOS One. Congratulations! Your manuscript is now being handed over to our production team.

At this stage, our production department will prepare your paper for publication. This includes ensuring the following:

* All references, tables, and figures are properly cited

* All relevant supporting information is included in the manuscript submission,

* There are no issues that prevent the paper from being properly typeset

You will receive further instructions from the production team, including instructions on how to review your proof when it is ready. Please keep in mind that we are working through a large volume of accepted articles, so please give us a few days to review your paper and let you know the next and final steps.

Lastly, if your institution or institutions have a press office, please let them know about your upcoming paper now to help maximize its impact. If they'll be preparing press materials, please inform our press team within the next 48 hours. Your manuscript will remain under strict press embargo until 2 pm Eastern Time on the date of publication. For more information, please contact onepress@plos.org.

You will receive an invoice from PLOS for your publication fee after your manuscript has reached the completed accept phase. If you receive an email requesting payment before acceptance or for any other service, this may be a phishing scheme. Learn how to identify phishing emails and protect your accounts at https://explore.plos.org/phishing.

If we can help with anything else, please email us at customercare@plos.org.

Thank you for submitting your work to PLOS ONE and supporting open access.

Kind regards,

PLOS ONE Editorial Office Staff

on behalf of

Dr. Petr Heneberg

Academic Editor

PLOS One
